# Dose and Time Dependencies in Stress Pathway Responses during Chemical Exposure: Novel Insights from Gene Regulatory Networks

**DOI:** 10.3389/fgene.2017.00142

**Published:** 2017-10-06

**Authors:** Terezinha M. Souza, Jos C. S. Kleinjans, Danyel G. J. Jennen

**Affiliations:** Department of Toxicogenomics, GROW School for Oncology and Developmental Biology, Maastricht University, Maastricht, Netherlands

**Keywords:** gene regulatory networks, network inference, toxicity pathways, hepatotoxicity, transcription networks

## Abstract

Perturbation of biological networks is often observed during exposure to xenobiotics, and the identification of disturbed processes, their dynamic traits, and dose–response relationships are some of the current challenges for elucidating the mechanisms determining adverse outcomes. In this scenario, reverse engineering of gene regulatory networks (GRNs) from expression data may provide a system-level snapshot embedded within accurate molecular events. Here, we investigate the composition of GRNs inferred from groups of chemicals with two distinct outcomes, namely carcinogenicity [azathioprine (AZA) and cyclophosphamide (CYC)] and drug-induced liver injury (DILI; diclofenac, nitrofurantoin, and propylthiouracil), and a non-carcinogenic/non-DILI group (aspirin, diazepam, and omeprazole). For this, we analyzed publicly available exposed *in vitro* human data, taking into account dose and time dependencies. Dose–Time Network Identification (DTNI) was applied to gene sets from exposed primary human hepatocytes using four stress pathways, namely endoplasmic reticulum (ER), NF-κB, NRF2, and TP53. Inferred GRNs suggested case specificity, varying in interactions, starting nodes, and target genes across groups. DILI and carcinogenic compounds were shown to directly affect all pathway-based GRNs, while non-DILI/non-carcinogenic chemicals only affected NF-κB. NF-κB-based GRNs clearly illustrated group-specific disturbances, with the cancer-related casein kinase *CSNK2A1* being a target gene only in the carcinogenic group, and opposite regulation of NF-κB subunits being observed in DILI and non-DILI/non-carcinogenic groups. Target genes in NRF2-based GRNs shared by DILI and carcinogenic compounds suggested markers of hepatotoxicity. Finally, we indicate several of these group-specific interactions as potentially novel. In summary, our reversed-engineered GRNs are capable of revealing dose dependent, chemical-specific mechanisms of action in stress-related biological networks.

## Introduction

In the last few years, investigation of biological processes disturbed by chemical exposure and its potential adverse effects has become the main goal in toxicological assessments targeting hazard identification and/or drug development. This strategy encompasses two steps: the first being the identification of such processes/pathways and the second, the estimation of dose–response, dynamic relationships that may define the boundaries between adaptive and adverse responses (Bhattacharya et al., 2011; Middleton et al., 2017).

Although a substantial amount of mechanistic information has been gained from applying high-throughput (HT) technologies (e.g., transcriptomics, proteomics, and metabolomics) to exposed *in vitro* models, the ability of current methods to address the aforementioned challenges has proven insufficient. Take, for instance, pathway analysis – omnipresent in HT studies as means to identify biological processes affected across different conditions. First, functional annotation does not reflect the diversity of the human genome, with the repertoire of pathways across multiple databases being either comprised of general processes (e.g., metabolism, signaling) or very specific responses (e.g., drug-related pathways) (Khatri et al., 2012). The choice of arbitrary thresholds for fold change and/or statistical significance, as well as stratification of the input gene list by direction of expression are an additional source of variation that influence the output qualitatively and quantitatively.

A more realistic portrayal potentially loaded with novel mechanistic insights can be achieved by reversely engineering gene regulatory networks (GRNs) using expression data; in contrast to pathways, GRNs are case specific, referencing multiple types of physical and biochemical interactions among genes and gene products (Madhamshettiwar et al., 2012), allowing more detailed investigations by not (or minimally) relying on prior knowledge. Previous investigations have attempted to extract novel biological information from GRNs, the majority focusing on the predictive value of (sub)networks and their potential use as biomarkers (Schadt, 2009; Emmert-Streib et al., 2014), which led to the discovery, for instance, of striking dissimilarities between networks of smokers with and without lung cancer (Wang et al., 2011). More than molecular snapshots of a specific phenotype, GRNs are important instruments to investigate the interface genotype–environment (i.e., diet, drugs, and chemical exposure). Early studies with *Saccharomyces cerevisiae* have shown that network interactions undergo critical changes after challenging with a DNA damaging agent, leading to extensive network rewiring (at least 70% out of 80,000 tested genetic interactions) (Bandyopadhyay et al., 2010). Other recent investigations employing low-throughput gene expression data further indicated that gene–gene interactions, although compound specific to some extent, show similar patterns resembling toxic properties across different chemicals – and incorporating interaction data into classification algorithms increase prediction accuracy (Yamane et al., 2016).

Recently, our research group has developed and validated a tool for inferring GRNs from HT gene expression data during chemical exposure, taking possible time and dose dependencies into account (Hendrickx et al., 2016). By using ordinary differential equations (ODEs), this method establishes a causal link between external perturbations and gene–gene interactions within a particular biological process, in addition to identifying potentially novel interactions. In this study, we aimed to extract mechanistic information from chemical-induced toxicity by reversely engineering GRNs using HT gene expression data. For this, we compare GRNs inferred from groups of chemicals with distinct adverse effects, namely carcinogenicity and drug-induced liver injury (DILI), to those generated by exposure to non-adverse compounds. Through the reconstruction of gene–gene interactions from four stress-related pathways (TP53, ER, NRF2, and NF-κB), we aimed to gather causal evidence for dose dependent, dynamic, and potentially novel biological information related to chemical exposure.

## Materials and Methods

### Dose–Time Network Identification (DTNI) Method

Dose–Time Network Identification (DTNI) is a method for inferring network interactions among genes through ODEs that relate changes in gene expression over time and dose and an external perturbation. DTNI requires measurements from multiple doses and time points – not necessarily sampled at equal intervals – and expression values of a reduced gene set (preferentially less than 100 genes). DTNI can be applied to single chemicals but also allows the use of group-wise approaches – in which a consensus network is inferred for multiple compounds. A detailed description of the method, its validation, and script availability for MATLAB is described elsewhere (Hendrickx et al., 2016).

### Chemical Selection

In order to link network changes to chemical effects, we targeted compounds with well-known (adverse) effects in humans. We opted for three groups, two with different mechanisms of toxicity – carcinogenicity and DILI – and one with no weight of evidence for human carcinogenicity or DILI. Since DTNI requires datasets with a minimum of three doses and three time points, we used the Japanese database TG-GATEs (Toxicogenomics Project-Genomics Assisted Toxicity Evaluation system^1^). TG-GATEs contains microarray data of hundreds of compounds, generated *in vitro* (human and rat) and *in vivo* (rat), tested at multiple doses and time points. We therefore mined TG-GATEs for compounds matching the criteria of full availability of sets (i.e., both replicates and all doses/time points) and specific classification regarding carcinogenicity or DILI. To also avoid methodological biases, we aimed to create groups with fairly equal number of compounds; based on these constraints, the carcinogenic group comprised two chemicals [azathioprine (AZA) and cyclophosphamide (CYC)], while DILI (diclofenac, propylthiouracil, and nitrofurantoin) and non-carcinogenic/non-DILI (diazepam, omeprazole, and aspirin) groups contained three chemicals each (**Table 1**). **Table 1** contains detailed information on chemicals and evidence for their inclusion in their respective groups.

**Table 1:** Description of chemical groups used to infer gene regulatory networks (GRNs).

Chemical abstracts service (CAS)	Group	Evidence for inclusion
Azathioprine (446-86-6)	Carcinogenic	Classified as carcinogenic to
Cyclophosphamide (6055-19-2)		humans by international agency for research on cancer (IARC)
Diclofenac (15307-86-5)	DILI	Use is associated with
Nitrofurantoin (67-20-9)		risk of acute liver injury^1^
Propylthiouracil (51-52-5)		
Aspirin (50-78-2)	Non-DILI/non-	Clinical cases of acute
Diazepam (439-14-5	carcinogenic	liver injury are very rare^1^
Omeprazole (73590-58-6)		Not classifiable or not classified as to its carcinogenicity by IARC


^1^*Information obtained from LiverTox database (livertox.nih.gov)*.

### Preprocessing of *In Vitro* Microarray Datasets

Raw files from each chemical set were downloaded from TG-GATEs^2^ and preprocessed (background correction, log2-base transformation, and normalization) through R scripts provided on ArrayAnalysis (arrayanalysis.org) (Eijssen et al., 2013). Probes were annotated using customCDF version 19 with Entrez identifiers. To obtain differentially expressed genes (DEGs), we used the R package LIMMA to perform moderated *t*-test comparing mean intensities from exposed and time-matched controls in all three doses tested. Detailed information from datasets used in this study (accession numbers from microarrays and respective compounds/dose/time points) is available on Supplementary Data 1.

### Selection of Biological Networks to Be Assessed by DTNI

Given our goal to identify common features underlying (non-)toxic mechanisms across chemicals, and DTNI’s requirement for reduced gene sets, we opted for a group-wise approach for evaluating how carcinogenic, DILI, and non-carcinogenic/non-DILI compounds may (differentially) affect networks involved in toxicological responses. Our selection involves known stress pathways indicative of DNA damage (TP53), oxidative stress (NRF2), endoplasmic reticulum (ER) stress, and inflammation (NF-κB). From KEGG, we retrieved gene components from pathways ER (166 genes, of which 158 were present in normalized sets), TP53 (69 genes, of which 68 were present in normalized sets), and NF-κB (94 genes, of which 89 were present in normalized sets) – entries hsa04141, hsa04115, and hsa04064, respectively. NRF2 genes (143, of which 126 were present in normalized sets) were obtained from WikiPathways (entry WP2884) (the list of genes for each pathway can be found in Supplementary Data 1).

To gain insights into the activity of these pathways in the groups tested, we performed an additional pathway analysis with all DEGs (FDR < 0.05 without fold change thresholds) from each chemical using the database ConsensusPathDB (CPDB) and its overrepresentation analysis tool (*q*-value < 0.05) (Kamburov et al., 2013).

### Network Inference of Selected Networks through DTNI

For DTNI, we used scripts developed for use on MATLAB (Hendrickx et al., 2016). For this, we used log2-transformed ratios from all chemicals within each group as input file. Then, we performed leave-one-out cross validation (LOOCV) by excluding data from one compound at a time before performing a new DTNI. In all cases, parameters from DTNI were left as default, with a threshold for interaction strength (*p*-value) set to 0.05. The final network for each pathway within a chemical group was obtained after determining the intersection among the DTNIs (i.e., all chemicals and LOOCVs). The consensus network for NF-κB in the DILI group, for instance, was generated after overlapping the results from DTNI with all three compounds plus LOOCVs (with a total of four different runs). Cytoscape was used to generate and visualize the networks.

### Comparison of GRNs across Groups of Chemicals: Biological Significance

To assess differences across networks generated by DTNI, we considered four aspects of the inferred GRNs: direct perturbations to the pathway being analyzed, number of interactions, genes involved, and overall direction of expression (up- or downregulation). Furthermore, while starting nodes (i.e., nodes with only outgoing interactions) may be used as basis to investigate potential molecular initiating events (MIEs), target genes may offer a clue to potential downstream effects. Therefore, those two categories of genes were investigated in more detail within the GRNs.

Furthermore, to validate the interactions found by our method, we used the “induced network modules” tool available on the database CPDB. For this, we used gene lists from each pathway as input. We allowed for intermediary nodes and limited our search to only high-confidence protein interactions in addition to genetic, biochemical, and gene regulatory ones. By comparing our inferred GRNs to those obtained from CPDB, we were able to detect indirect, direct, and potentially novel interactions. A predicted interaction was labeled “direct” when the same edge was present in CPDB and “indirect” when a third node mediated an interaction between two nodes. Indirect interactions may appear due to reactions that happen faster than the timescale measured (e.g., minutes instead of hours) (Hendrickx et al., 2016). Potentially novel interactions were identified as direct interactions predicted by DTNI and not present in CPDB.

## Results

### Inferred GRNs Vary across Groups of Chemicals with Different Toxicities: Direct Perturbations and Number of Interactions

GRNs reconstructed from gene expression data showed strong variation across carcinogenic, DILI, and non-carcinogenic/non-DILI groups. The number of significant edges (*p*-value < 0.05) found for each pathway in each chemical group is summarized in **Table 2**. DILI and carcinogenic compounds affected all networks tested, with the former frequently resulting in the highest number of gene interactions. In contrast, non-carcinogenic/non-DILI compounds only affected NF-κB genes. When generating the networks, we observed that those from DILI are more connected in comparison with the other groups. TP53 and ER resulted in sets of unconnected modules in the carcinogenic group. An overview of such network layouts is represented in Supplementary Data 1.

**Table 2:** Number of edges obtained after applying DTNI to gene expression data from primary human hepatocytes exposed to chemicals with distinct toxicity.

Pathway	Carcinogenic	DILI	Non-carcinogenic/non-DILI
ER	22	57	0
NF-κB	20	39	39
NRF2	59	29	0
TP53	8	94	0


*Carcinogenic: azathioprine and cyclophosphamide, DILI: diclofenac, propylthiouracil, and nitrofurantoin, Non-carcinogenic/non-DILI: diazepam, omeprazole, and aspirin*.

The aforementioned results contrast with those from pathway analysis, which showed that all groups of chemicals affected biological pathways associated with P53 and NRF2 (**Table 3** and Supplementary Data 1). Protein processing in ER was a hit for both Carcinogenic and DILI groups, while NF-κB was significant for non-DILI/non-carcinogenic and DILI groups.

**Table 3:** Number of distinct biological processes affected by groups of chemicals related to investigated pathways – results from overrepresentation analysis using differentially expressed genes.

Pathway	Carcinogenic	DILI	Non-carcinogenic/non-DILI
ER	1	1	0
NF-κB	0	2	2
NRF2	3	3	3
TP53	4	4	3


*Carcinogenic: azathioprine and cyclophosphamide, DILI: diclofenac, propylthiouracil, and nitrofurantoin, Non-carcinogenic/non-DILI: diazepam, omeprazole, and aspirin*.

### Gene Disturbances in GRNs across Chemical Groups: Target Genes and Starting Nodes

**FIGURE 1:**
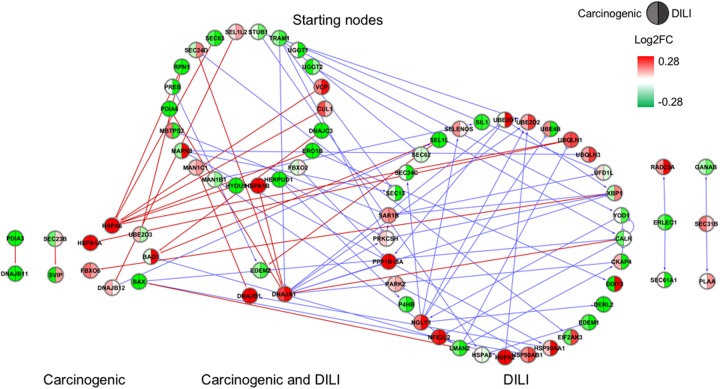
Gene regulatory network (GRN) inferred for members from the endoplasmic reticulum (ER) pathway. Red-colored edges: interactions predicted for carcinogenic group; Blue-colored edges: interactions predicted for drug-induced liver injury (DILI) group. Cluster in the upper section indicate starting nodes, while clusters in the lower section are comprised of target genes specific to each chemical group.

**FIGURE 2:**
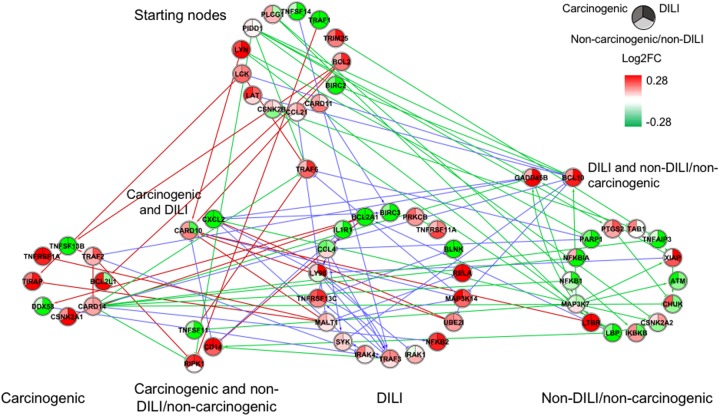
Gene regulatory network (GRN) inferred for members from the NF-κB pathway. Red-colored edges: interactions predicted for carcinogenic group; Blue-colored edges: interactions predicted for drug-induced liver injury (DILI) group; Green-colored edges: interactions predicted for non-DILI/non-carcinogenic group. Cluster in the upper section indicate starting nodes, while clusters in the lower section are comprised of target genes specific to each chemical group.

**FIGURE 3:**
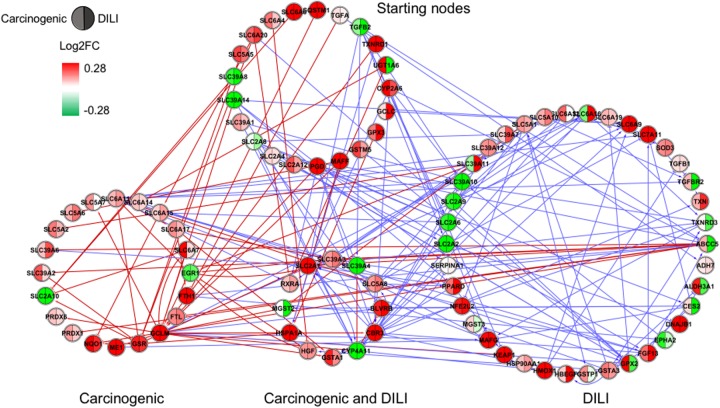
Gene regulatory network (GRN) inferred for members from the NRF2 pathway. Red-colored edges: interactions predicted for carcinogenic group; Blue-colored edges: interactions predicted for drug-induced liver injury (DILI) group. Cluster in the upper section indicate starting nodes, while clusters in the lower section are comprised of target genes specific to each chemical group.

**FIGURE 4:**
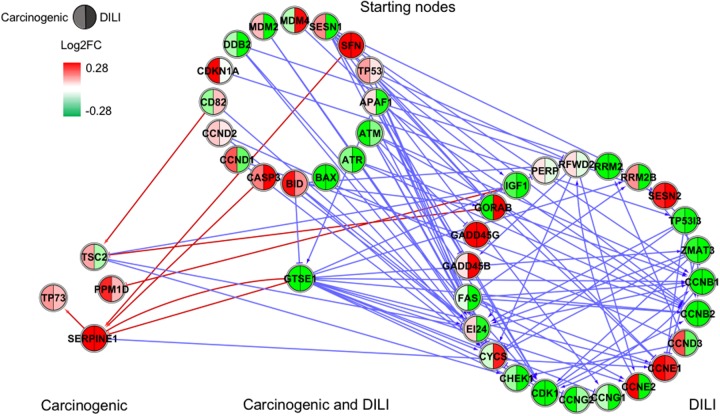
Gene regulatory network (GRN) inferred for members from the TP53 pathway. Red-colored edges: interactions predicted for carcinogenic group; Blue-colored edges: interactions predicted for drug-induced liver injury (DILI) group. Cluster in the upper section indicate starting nodes, while clusters in the lower section are comprised of target genes specific to each chemical group.

Although input gene lists were identical, the resulting inferred networks showed great variation across chemical groups. To enable visualization and direct comparison, pathways were represented as the union of all interactions predicted in each chemical group, in which edge colors express different chemical groups (red for carcinogenic, blue for DILI, and green for non-DILI/non-carcinogenic) (**Figures 1–4**).

First, we evaluated similarities in interactions from inferred networks, and observed that only NF-κB and NRF2 showed an overlap of two-node interactions in different groups. A negative interaction between *CARD14* and *BCL10* was detected in NF-κB for both DILI and non-DILI/non-carcinogenic groups. For NRF2, we found the negative interactions *GCLM*-*CYP4A11* and *ABCC5*-*SLC2A1* shared by DILI and carcinogenic groups.

Since only a few interactions were shared among all groups, we decided to investigate whether this low overlap was due to distinct starting nodes and/or target genes. These are indicated as different sections in the networks, with the upper part containing starting nodes and the lower part the target genes; the latter is further divided into clusters to highlight genes that are specific or shared by more than one chemical group. This arrangement allowed us to detect striking differences among groups. First, we observed that the only pathway that resulted in shared target genes among all three groups was NF-κB – *TRAF6*, a gene from the TNF receptor associated factor family. Also for NF-κB, some genes were also shared by DILI and non-DILI/non-carcinogenic group (*GADD45B* and *BCL10*), as well as DILI and carcinogenic (*CXCL12* and *CARD10*), and carcinogenic and non-DILI/non-carcinogenic (*TNFSF11*, *RIPK1* and *CD14*). NRF2 showed the highest overlap, with 12 genes shared between DILI and carcinogenic compounds: *BLVRB, SLC39A4, HGF, SLC39A3, CBR3, CYP4A11, GSTA1, HSPA1A, SLC2A1, MGST2, SLC5A8*, and *RXRA. GTSE1* was the only gene shared between DILI and carcinogenic chemicals for the TP53 pathway, while *EDEM2*, *DNJB1*, and *DNJA1*, from the ER pathway, were found shared by the aforementioned groups.

Overall, we also detected distinct starting nodes, frequently targeting specific clusters of chemical groups, suggesting that these potential MIEs may result in different responses within the same pathway.

### Gene Disturbances in GRNs across Chemical Groups: Validation of Predicted Interactions and Direction of Expression

To validate the edges predicted by DTNI, we compared our results to databases sourcing protein, genetic, and gene regulatory interactions as well as biochemical reactions. The detailed list of edges analyzed is provided in Supplementary Data 1. At least 40 and 30% of all edges predicted for TP53 and NF-κB, respectively, were present in CPDB as direct or indirect interactions. The majority of edges in NRF2 and ER sets, on the other hand, were labeled as potentially novel.

Since direction of expression is also an important feature to understand activation/repression of downstream biological effects, we added expression values to network nodes representing the average log2-transformed ratio of each gene. For cross-group comparisons, we partitioned every node into two or three segments, each containing the average expression calculated for the highest dose and latest time point – representing the maximum response across all measurements (**Figures 1–4**). With that, we aimed to understand the regulation of such processes during exposure. Also, by generating individual chemical group/network graphics interchange format files, we confirmed these biological interactions as time- and dose dependent, showing clear oscillatory and/or progressive gene expression profiles (Supplementary Data 2).

In general, we observed great differences in gene regulation across chemical groups, especially in group-specific clusters. Shared genes, on the other hand, usually showed the same direction of expression with variable intensities. Considering global expression, we observed that most genes from TP53 pathway were downregulated after exposure to DILI compounds; the same was observed for NF-κB inferred for non-DILI/non-carcinogenic group. Both therefore suggest repression of these processes. On the other hand, widespread activation of NRF2 was indicated by upregulation of most genes in both carcinogenic and DILI groups, although solute carrier genes *SLC2A2, SLC2A6, SLC2A9, SLC39A10*, found to be target genes only in DILI group, were repressed.

## Discussion

The main vision for modern toxicity testing proposes a shift from apical measurements (i.e., pathological modifications related to a disease state) in non-human *in vivo* models toward HT approaches *in vitro*. In the present study, we try to address this challenge with a systematic approach using DTNI, an *in silico* tool that models gene expression changes taking into account dose and time dependencies during chemical exposure.

To investigate the behavior of pathway activation in exposed models, we investigated the effects of chemicals with different adverse outcomes (acute organ injury and carcinogenicity) compared to drugs not implicated in these pathologies. We aimed to reconstruct pathways based on gene expression data; for this, we selected four pathways (NRF2, ER, TP53, and NF-κB) due to their established involvement in mechanisms of toxicity (Jennings, 2013; Jennings et al., 2013). Our first observation was that pathway hits did not relate to direct perturbations to these networks. For instance, even though TP53 and NRF2 were significant in all groups of chemicals, only DILI and carcinogenic compounds showed direct effect (i.e., activation) on these networks. On the other hand, direct perturbation to the NF-κB pathway was only observed for non-DILI/non-carcinogenic compounds. Interestingly, the inhibition of NF-κB, in line of our observation of overall repression of these genes, was confirmed to two components of this group, aspirin and diazepam. Therefore, it seems that in contrast to pathway hits, which may reflect indirect effects or common causes, establishing a causal, direct link between exposure and network perturbations may offer more accurate evidence for the mechanisms of action (Woo et al., 2015).

In addition to different levels of perturbation, we also noticed the distinguishing aspects of GRN composition among groups. This confirms previous findings on the dynamic traits of biological networks, where noticeable rewiring was observed between different disease phenotypes (Mani et al., 2008) and following chemical exposure (Bandyopadhyay et al., 2010), with both studies concluding that genetic interactions may be condition dependent. This was evident in our inferred GRNs, and we suggest that such dissimilarities may be due to distinct initial perturbations, which in turn lead to alternative routes within the same pathway. Our results show that edge paths traced from starting nodes, as well as clusters of target genes, are mostly group specific (**Figures 1–4**). This can be illustrated by the GRNs inferred for genes from the NF-κB pathway (**Figure 2**). Although all groups directly affected this process, GRNs indicate that while non-DILI/non-carcinogenic compounds targeted the inhibition of NF-KB1 and NF-KBIA, those from the DILI group resulted in activation of NF-KB2 and RELA. In addition to differences in direction of expression of these targets, studies have also shown that some NF-κB units may act independently from each other, controlling proliferation and immune responses (Ishikawa et al., 1997, 1998). Therefore, investigation of these branches of biological events may be crucial to differentiate adverse from non-adverse outcomes.

Besides these widespread differences, we also detected some similarities. GRNs inferred for genes from the NRF2 pathway resulted in the highest overlap of target genes with consistent direction of expression between carcinogenic and DILI groups (**Figure 3**). Among these genes, we identified hepatocyte growth factor (*HGF*), known to play a role in tumorigenesis and tissue regeneration (Huh et al., 2004). Recent studies have shown *HGF* to play a role in acute liver injury, by protecting against isoniazid- and rifampicin-oxidative liver damage (Enriquez-Cortina et al., 2013). Another interesting target shared by both groups was enzyme biliverdin reductase B (*BLVRB*), the gene coding, which converts biliverdin to bilirubin. Bilirubin, which has long been regarded as a cytotoxic waste product of heme metabolism, was recently discovered to possess strong antioxidant activity (Stocker et al., 1987). *CBR3*, a gene coding for an enzyme involved in the biotransformation of carbonyl compounds, was shown to be involved in predisposition of toxic responses in doxorubicin-treated patients (Fan et al., 2008). Taken together, these findings may also suggest that, in an attempt to avoid extreme injury, cells try to compensate by eliciting potent responders; because these mechanisms also have deleterious effects, shifts in their equilibrium may be the tipping point between adaptive and adverse responses.

Interestingly, NRF2 seemed to be the only GRN denoting similar toxic effects in DILI and carcinogenic groups. Perturbations in ER and TP53 pathways by chemicals in the carcinogenic group were very limited (**Figures 1, 4**), comprised mostly by fragmented subnetworks. Since carcinogens AZA and CYC also share DNA-damaging properties, we expected a large amount of disturbances in GRNs from both pathways. In view of this, we hypothesize that due to the different mechanisms underlying their effects (CYC is an alkylating agent while AZA is an antimetabolite), common significant interactions could not be inferred from the available data. The fact that these chemicals have stronger effects on dividing cells may yet be another cause, since human hepatocytes show limited proliferative capacity when cultured (Ramboer et al., 2014).

More importantly, many of the interactions found in our study were labeled as potentially novel ones. During our validation step, we noticed that interactions annotated for the NRF2 pathway in particular were very limited in comparison with the other three (only 672 against 1518, 1609, and 1539 in ER, NF-κB, and TP53, respectively). The fact that TP53 and NF-κB have been extensively studied over the years due to their clinical relevance may explain why they showed the highest number of direct or indirect positive hits (more than 40 and 30% of all inferred interactions, respectively). Therefore, our results indicate the need for further studies targeting the validation of such interactions as means to expand the repertoire of biological interactions and assess their relevance as potential markers of toxicity.

It should be pointed out some of the limitations of GRN methods, in particular ODE-based methods such as DTNI. Although ODE-based methodology describes time-series data well, its deterministic nature does not account for statistical fluctuations in concentrations and kinetic parameters that greatly influence biological systems (Palmer and Shearwin, 2009). The generation of a consensus network using multiple compounds implemented in DTNI partly solves the problem of data availability, but also constrains chemical selection to substances with somewhat similar effects whose interactions survive after steps of LOOCV. It may also result in increased computational times if a large number of chemicals and input genes are being assessed.

Nonetheless, our approach demonstrated some unprecedented mechanistic aspects of GRNs upon exposure to chemicals with different toxic potential. First, GRNs are usually condition dependent, indicating distinct molecular events depending on the type of exposure. To some extent, however, there are similar gene targets shared by GRNs inferred for toxic groups – but not present in compounds considered non-DILI/non-carcinogenic – that may point toward relevant molecular events indicative of toxicity. Finally, the fact that disturbances in these molecular targets evolve with increases in dose reinforces the value of DTNI as an asset in network-based, HT investigations. Anchoring these dose-dependent events to apical measurements may therefore reveal molecular signatures and clarify the tipping points leading to adverse outcomes.

## Author Contributions

TS analyzed the data and wrote the manuscript. JK and DJ wrote the manuscript.

## Conflict of Interest Statement

The authors declare that the research was conducted in the absence of any commercial or financial relationships that could be construed as a potential conflict of interest. The reviewer KG and handling Editor declared their shared affiliation.

## Footnotes

1 toxico.nibiohn.go.jp/english/

2 toxico.nibiohn.go.jp/
