# Supplementary material for: Dose and Time Dependencies in Stress Pathway Responses during Chemical Exposure: Novel Insights from Gene Regulatory Networks
Source: Front Genet. 2017 Oct 6;8:142. doi: 10.3389/fgene.2017.00142 (PMC5649202; doi:10.3389/fgene.2017.00142)
Supplement: Supplementary file 1 [file Table_1.PDF]

Supplementary Data 1

Contents

1. Detailed information on arrays used in this study – ArrayExpress study accession E-MTAB-798 2

Carcinogenic 2

DILI.....3

Non-carcinogenic/non-DILI 4

2. Biological processes affected by exposure to chemicals studied related to pathways investigated. Differentially expressed genes (FDR <0.05, FC > 1.5) were used as input for the over-representation tool from available on the database CPDB. 5

3. Genes used as input for DTNI, per pathway analyzed 8

ER - KEGG accession hsa04141 8

Nrf2 - Wikipathways accession WP2884 9

NF-KB - KEGG accession hsa04064 10

TP53 - KEGG accession hsa04115 11

4. Overview of networks inferred for each pathway by DTNI, per chemical group. 12

5. Validation of edges predicted by DTNI using annotated interactions available on CPDB. 13

a. NRF2 13

b. NF-KB 21

c. ER 26

d. TP53 30

6. Investigation from novel interactions in NRF2 pathway: STRING results 34

a. CBR3 34

b. HGF 35

.....35

c. BLVRB 36

1. Detailed information on arrays used in this study – ArrayExpress study accession **E-MTAB-798**

Carcinogenic			
**Azathioprine**			
	**2hr**	**8hr**	**24hr**
**Control**	003016044009.CEL	003016044017.CEL	003016044025.CEL
	003016044010.CEL	003016044018.CEL	003016044026.CEL
**Low**	003016044011.CEL	003016044019.CEL	003016044027.CEL
	003016044012.CEL	003016044020.CEL	003016044028.CEL
**Middle**	003016044013.CEL	003016044021.CEL	003016044029.CEL
	003016044014.CEL	003016044022.CEL	003016044030.CEL
**High**	003016044015.CEL	003016044023.CEL	003016045001.CEL
	003016044016.CEL	003016044024.CEL	003016045002.CEL

**Cyclophosphamide**			
	**2hr**	**8hr**	**24hr**
**Control**	003016035006.CEL	003016035014.CEL	003016035022.CEL
	003016035007.CEL	003016035015.CEL	003016035023.CEL
**Low**	003016035008.CEL	003016035016.CEL	003016035024.CEL
	003016035009.CEL	003016035017.CEL	003016035025.CEL
**Middle**	003016035010.CEL	003016035018.CEL	003016035026.CEL
	003016035011.CEL	003016035019.CEL	003016035027.CEL
**High**	003016035012.CEL	003016035020.CEL	003016035028.CEL
	003016035013.CEL	003016035021.CEL	003016035029.CEL

DILI			
**Nitrofurantoin**			
	**2hr**	**8hr**	**24hr**
**Control**	003016030013.CEL	003016030021.CEL	003016030029.CEL
	003016030014.CEL	003016030022.CEL	003016030030.CEL
**Low**	003016030015.CEL	003016030023.CEL	003016048009.CEL
	003016030016.CEL	003016030024.CEL	003016048010.CEL
**Middle**	003016030017.CEL	003016030025.CEL	003016048011.CEL
	003016030018.CEL	003016030026.CEL	003016048012.CEL
**High**	003016030019.CEL	003016030027.CEL	003016031005.CEL
	003016030020.CEL	003016030028.CEL	003016031006.CEL

Diclofenac			
	**2hr**	**8hr**	**24hr**
**Control**	003016038015.CEL	003016038023.CEL	003016039001.CEL
	003016038016.CEL	003016038024.CEL	003016039002.CEL
**Low**	003016038017.CEL	003016038025.CEL	003016039003.CEL
	003016038018.CEL	003016038026.CEL	003016039004.CEL
**Middle**	003016038019.CEL	003016038027.CEL	003016039005.CEL
	003016038020.CEL	003016038028.CEL	003016039006.CEL
**High**	003016038021.CEL	003016038029.CEL	003016039007.CEL
	003016038022.CEL	003016038030.CEL	003016039008.CEL

Propylthiouracil			
	**2hr**	**8hr**	**24hr**
**Control**	003016039009.CEL	003016039017.CEL	003016039025.CEL
	003016039010.CEL	003016039018.CEL	003016039026.CEL
**Low**	003016039011.CEL	003016039019.CEL	003016039027.CEL
	003016039012.CEL	003016039020.CEL	003016039028.CEL
**Middle**	003016039013.CEL	003016039021.CEL	003016039029.CEL
	003016039014.CEL	003016039022.CEL	003016039030.CEL
**High**	003016039015.CEL	003016039023.CEL	003016040001.CEL
	003016039016.CEL	003016039024.CEL	003016040002.CEL

Non-carcinogenic/non-DILI			
	**Aspirin**		
	**2hr**	**8hr**	**24hr**
**Control**	003016024019.CEL	003016024027.CEL	003016025005.CEL
	003016024020.CEL	003016024028.CEL	003016025006.CEL
**Low**	003016024021.CEL	003016024029.CEL	003016025007.CEL
	003016024022.CEL	003016024030.CEL	003016025008.CEL
**Middle**	003016024023.CEL	003016025001.CEL	003016025009.CEL
	003016024024.CEL	003016025002.CEL	003016025010.CEL
**High**	003016024025.CEL	003016025003.CEL	003016025011.CEL
	003016024026.CEL	003016025004.CEL	003016025012.CEL

	**Diazepam**		
	**2hr**	**8hr**	**24hr**
**Control**	003016031007.CEL	003016031015.CEL	003016031023.CEL
	003016031008.CEL	003016031016.CEL	003016031024.CEL
**Low**	003016031009.CEL	003016031017.CEL	003016031025.CEL
	003016031010.CEL	003016031018.CEL	003016031026.CEL
**Middle**	003016031011.CEL	003016031019.CEL	003016031027.CEL
	003016031012.CEL	003016031020.CEL	003016031028.CEL
**High**	003016031013.CEL	003016031022.CEL	003016031029.CEL
	003016031014.CEL	003016041027.CEL	003016031030.CEL

	**Omeprazole**		
	**2hr**	**8hr**	**24hr**
**Control**	003017026016.CEL	003017026024.CEL	003017027002.CEL
	003017026017.CEL	003017026025.CEL	003017027003.CEL
**Low**	003017026018.CEL	003017026026.CEL	003017027004.CEL
	003017026019.CEL	003017026027.CEL	003017027005.CEL
**Middle**	003017026020.CEL	003017026028.CEL	003017027006.CEL
	003017026021.CEL	003017026029.CEL	003017027007.CEL
**High**	003017026022.CEL	003017026030.CEL	003017027008.CEL
	003017026023.CEL	003017027001.CEL	003017027009.CEL

2. Biological processes affected by exposure to chemicals studied related to pathways investigated. Differentially expressed genes (FDR <0.05, FC > 1.5) were used as input for the over-representation tool from available on the database CPDB.

NRF2	Carcinogenic	**Azathioprine**	**Cyclophosphamide**	**-**
		oxidative stress induced gene expression via nrf2	Transcriptional activation by NRF2	-
		Transcriptional activation by NRF2	-	-
		NRF2 pathway	-	-
	DILI	**Diclofenac**	**Nitrofurantoin**	**Propylthiouracil**
		NRF2 pathway	oxidative stress induced gene expression via nrf2	NRF2 pathway
		oxidative stress induced gene expression via nrf2	Transcriptional activation by NRF2	oxidative stress induced gene expression via nrf2
			NRF2 pathway	-
	Non-DILI/Non-carcinogenic	**Aspirin**	**Diazepam**	**Omeprazole**
		**-**	oxidative stress induced gene expression via nrf2	Transcriptional activation by NRF2
		-	Transcriptional activation by NRF2	oxidative stress induced gene expression via nrf2
		-	NRF2 pathway	NRF2 pathway
TP53	Carcinogenic	**Azathioprine**	**Cyclophosphamide**	**-**
		p53 signaling pathway - Homo sapiens (human)	p53 signaling pathway - Homo sapiens (human)	-
		Direct p53 effectors	p53-Dependent G1 DNA Damage Response	-
		p53-Dependent G1 DNA Damage Response	p53-Dependent G1/S DNA damage checkpoint	-
		p53-Dependent G1/S DNA damage checkpoint	-	-

	**DILI**	**Diclofenac**	**Nitrofurantoin**	**Propylthiouracil**
		p53 signaling pathway - Homo sapiens (human)	p53 signaling pathway - Homo sapiens (human)	Direct p53 effectors
		-	Direct p53 effectors	p53 signaling pathway - Homo sapiens (huma
		-	-	p53 pathway
	**Non-DILI/Non-carcinogenic**	**Aspirin**	**Diazepam**	**Omeprazole**
		Direct p53 effectors	Direct p53 effectors	Direct p53 effectors
		p53 signaling pathway - Homo sapiens (human)	p53 signaling pathway - Homo sapiens (human)	p53 signaling pathway - Homo sapiens (human)
		-	p53 signaling pathway	-
**ER**	**Carcinogenic**	**Azathioprine**	**Cyclophosphamide**	**-**
		Protein processing in endoplasmic reticulum - Homo sapiens (human)	-	-
	**DILI**	**Diclofenac**	**Nitrofurantoin**	**Propylthiouracil**
		**-**	Protein processing in endoplasmic reticulum - Homo sapiens (human)	-
	**Non-DILI/Non-carcinogenic**	**Aspirin**	**Diazepam**	**Omeprazole**
**NF-KB**	**Carcinogenic**	**Azathioprine**	**Cyclophosphamide**	**-**
		Protein processing in endoplasmic reticulum - Homo sapiens (human)	-	-
	**DILI**	**Diclofenac**	**Nitrofurantoin**	**Propylthiouracil**
		**-**	TNF receptor superfamily (TNFSF) members mediating non-canonical NF-kB pathway	Quercetin and Nf-kB- AP-1 Induced Cell Apoptosis

	Aspirin	Diazepam	Omeprazole
**Non-DILI/Non-carcinogenic**	**-**	Quercetin and Nf-kB- AP-1 Induced Cell Apoptosis	Quercetin and Nf-kB- AP-1 Induced Cell Apoptosis
	-	-	NF-kB activation through FADD/RIP-1 pathway mediated by caspase-8 and -10

3. Genes used as input for DTNI, per pathway analyzed

ER - KEGG accession hsa04141															
Gene ID	Gene Symbol	Gene ID	Gene Symbol	Gene ID	Gene Symbol	Gene ID	Gene Symbol	Gene ID	Gene Symbol	Gene ID	Gene Symbol	Gene ID	Gene Symbol	Gene ID	Gene Symbol
267	AMFR	3309	HSPA5	5887	RAD23B	7494	XBP1	10483	SEC23B	26232	FBXO2	55768	NGLY1	258010	SVIP
573	BAG1	3310	HSPA6	6184	RPN1	7841	MOGS	10484	SEC23A	26270	FBXO6	55829	SELENOS	285126	DNAJC5G
581	BAX	3312	HSPA8	6185	RPN2	7991	TUSC3	10525	HYOU1	27102	EIF2AK1	55968	NSFL1C	440275	EIF2AK4
596	BCL2	3320	HSP90AA1	6238	RRBP1	8454	CUL1	10802	SEC24A	27248	ERLEC1	56605	ERO1B	100506742	CASP12
811	CALR	3326	HSP90AB1	6396	SEC13	8720	MBTPS1	10808	HSPH1	29927	SEC61A1	56681	SAR1A		
821	CANX	3337	DNAJB1	6400	SEL1L	9373	PLAA	10905	MAN1A2	29978	UBQLN2	56886	UGGT1		
823	CAPN1	3703	STT3A	6500	SKP1	9451	EIF2AK3	10952	SEC61B	29979	UBQLN1	57134	MAN1C1		
824	CAPN2	3998	LMAN1	6745	SSR1	9532	BAG2	10956	OS9	30001	ERO1A	64215	DNAJC1		
1388	ATF6B	4121	MAN1A1	6746	SSR2	9601	PDIA4	10960	LMAN2	50613	UBQLN3	64374	SIL1		
1410	CRYAB	4217	MAP3K5	6747	SSR3	9632	SEC24C	10961	ERP29	51009	DERL2	79139	DERL1		
1603	DAD1	4287	ATXN3	6748	SSR4	9695	EDEM1	10970	CKAP4	51128	SAR1B	79748	LMAN1L		
1649	DDIT3	4780	NFE2L2	7095	SEC62	9709	HERPUD1	11231	SEC63	51360	MBTPS2	80267	EDEM3		
1650	DDOST	5034	P4HB	7184	HSP90B1	9871	SEC24D	11253	MAN1B1	51465	UBE2J1	80331	DNAJC5		
1965	EIF2S1	5071	PRKN	7186	TRAF2	9978	RBX1	22824	HSPA4L	51619	UBE2D4	80343	SEL1L2		
2081	ERN1	5589	PRKCSH	7321	UBE2D1	10113	PREB	22872	SEC31A	51726	DNAJB11	80700	UBXN6		
2923	PDIA3	5599	MAPK8	7322	UBE2D2	10130	PDIA6	22926	ATF6	54431	DNAJC10	84447	SYVN1		
3300	DNAJB2	5601	MAPK9	7323	UBE2D3	10134	BCAP31	23193	GANAB	54788	DNAJB12	85479	DNAJC5B		
3301	DNAJA1	5602	MAPK10	7326	UBE2G1	10273	STUB1	23471	TRAM1	55176	SEC61A2	91319	DERL3		
3303	HSPA1A	5609	MAP2K7	7327	UBE2G2	10277	UBE4B	23480	SEC61G	55432	YOD1	92552	ATXN3L		
3304	HSPA1B	5610	EIF2AK2	7353	UFD1	10294	DNAJA2	23640	HSPBP1	55666	NPLOC4	118424	UBE2J2		
3305	HSPA1L	5611	DNAJC3	7415	VCP	10299	MARCH6	23645	PPP1R15A	55741	EDEM2	143630	UBQLNL		
3306	HSPA2	5886	RAD23A	7466	WFS1	10427	SEC24B	25956	SEC31B	55757	UGGT2	201595	STT3B		

Nrf2 - Wikipathways accession WP2884

Gene ID	Gene Symbol	Gene ID	Gene ID	Gene Symbol	Gene ID	Gene ID	Gene Symbol	Gene ID	Gene Symbol	Gene ID	Gene Symbol
131	ADH7	2938	GSTA1	5467	PPARD	7039	TGFA	25800	SLC39A6	159963	SLC5A12
177	AGER	2940	GSTA3	6256	RXRA	7040	TGFB1	27173	SLC39A1	160728	SLC5A8
218	ALDH3A1	2941	GSTA4	6513	SLC2A1	7042	TGFB2	28968	SLC6A16	200010	SLC5A9
645	BLVRB	2947	GSTM3	6514	SLC2A2	7048	TGFBR2	29985	SLC39A3	201266	SLC39A11
873	CBR1	2948	GSTM4	6515	SLC2A3	7295	TXN	29986	SLC39A2	221074	SLC39A12
874	CBR3	2949	GSTM5	6517	SLC2A4	7296	TXNRD1	29988	SLC2A8	221223	CES5A
1244	ABCC2	2950	GSTP1	6518	SLC2A5	7922	SLC39A7	54578	UGT1A6	283375	SLC39A5
1548	CYP2A6	3082	HGF	6523	SLC5A1	8714	ABCC3	54716	SLC6A20	283848	CES4A
1579	CYP4A11	3084	NRG1	6524	SLC5A2	8824	CES2	55117	SLC6A15	340024	SLC6A19
1728	NQO1	3162	HMOX1	6526	SLC5A3	8878	SQSTM1	55334	SLC39A9	348932	SLC6A18
1839	HBEGF	3303	HSPA1A	6527	SLC5A4	8884	SLC5A6	55630	SLC39A4	388662	SLC6A17
1958	EGR1	3320	HSP90AA1	6528	SLC5A5	9152	SLC6A5	56606	SLC2A9		
1969	EPHA2	3326	HSP90AB1	6529	SLC6A1	9588	PRDX6	57181	SLC39A10		
2042	EPHA3	3337	DNAJB1	6530	SLC6A2	9817	KEAP1	60482	SLC5A7		
2258	FGF13	4097	MAFG	6531	SLC6A3	10057	ABCC5	64116	SLC39A8		
2495	FTH1	4199	ME1	6532	SLC6A4	10257	ABCC4	66035	SLC2A11		
2512	FTL	4258	MGST2	6533	SLC6A6	11182	SLC2A6	81031	SLC2A10		
2539	G6PD	4259	MGST3	6534	SLC6A7	11254	SLC6A14	91252	SLC39A13		
2729	GCLC	4780	NFE2L2	6535	SLC6A8	22949	PTGR1	114112	TXNRD3		
2730	GCLM	5052	PRDX1	6536	SLC6A9	23491	CES3	114134	SLC2A13		
2877	GPX2	5155	PDGFB	6538	SLC6A11	23516	SLC39A14	115584	SLC5A11		
2878	GPX3	5226	PGD	6540	SLC6A13	23657	SLC7A11	125206	SLC5A10		
2936	GSR	5265	SERPINA1	6649	SOD3	23764	MAFF	154091	SLC2A12		

NF-KB - KEGG accession hsa04064									
Gene ID	Gene Symbol	Gene ID	Gene Symbol	Gene ID	Gene Symbol	Gene ID	Gene Symbol	Gene ID	Gene Symbol
142	PARP1	3654	IRAK1	6363	CCL19	8792	TNFRSF11A	257397	TAB3
329	BIRC2	3929	LBP	6366	CCL21	8837	CFLAR		
330	BIRC3	3932	LCK	6387	CXCL12	8915	BCL10		
331	XIAP	4049	LTA	6850	SYK	9020	MAP3K14		
472	ATM	4050	LTB	6885	MAP3K7	10454	TAB1		
596	BCL2	4055	LTBR	7099	TLR4	10673	TNFSF13B		
597	BCL2A1	4067	LYN	7124	TNF	10892	MALT1		
598	BCL2L1	4615	MYD88	7128	TNFAIP3	23085	ERC1		
695	BTK	4616	GADD45B	7132	TNFRSF1A	23118	TAB2		
929	CD14	4790	NFKB1	7185	TRAF1	23586	DDX58		
958	CD40	4791	NFKB2	7186	TRAF2	23643	LY96		
959	CD40LG	4792	NFKBIA	7187	TRAF3	27040	LAT		
1147	CHUK	5328	PLAU	7188	TRAF5	29760	BLNK		
1457	CSNK2A1	5335	PLCG1	7189	TRAF6	29775	CARD10		
1459	CSNK2A2	5336	PLCG2	7329	UBE2I	51135	IRAK4		
1460	CSNK2B	5579	PRKCB	7412	VCAM1	51588	PIAS4		
2920	CXCL2	5588	PRKCQ	7535	ZAP70	55367	PIDD1		
3383	ICAM1	5743	PTGS2	7706	TRIM25	79092	CARD14		
3551	IKBKB	5970	RELA	8600	TNFSF11	84433	CARD11		
3553	IL1B	5971	RELB	8717	TRADD	114609	TIRAP		
3554	IL1R1	6351	CCL4	8737	RIPK1	115650	TNFRSF13C		
3576	CXCL8	6357	CCL13	8740	TNFSF14	148022	TICAM1		

TP53 - KEGG accession hsa04115					
Gene ID	Gene Symbol	Gene ID	Gene Symbol	Gene ID	Gene Symbol
317	APAF1	1111	CHEK1	9134	CCNE2
355	FAS	1643	DDB2	9538	EI24
472	ATM	1647	GADD45A	9540	TP53I3
545	ATR	2810	SFN	10912	GADD45G
575	ADGRB1	3479	IGF1	25898	RCHY1
581	BAX	3486	IGFBP3	27113	BBC3
595	CCND1	3732	CD82	27244	SESN1
637	BID	4193	MDM2	50484	RRM2B
836	CASP3	4194	MDM4	51246	SHISA5
841	CASP8	4616	GADD45B	51512	GTSE1
842	CASP9	5054	SERPINE1	54205	CYCS
891	CCNB1	5268	SERPINB5	55240	STEAP3
894	CCND2	5366	PMAIP1	55367	PIDD1
896	CCND3	5728	PTEN	56475	RPRM
898	CCNE1	6241	RRM2	63970	TP53AIP1
900	CCNG1	6477	SIAH1	64065	PERP
901	CCNG2	7057	THBS1	64326	RFWD2
983	CDK1	7157	TP53	64393	ZMAT3
1017	CDK2	7161	TP73	83667	SESN2
1019	CDK4	7249	TSC2	85417	CCNB3
1021	CDK6	8493	PPM1D	92344	GORAB
1026	CDKN1A	8795	TNFRSF10B	143686	SESN3
1029	CDKN2A	9133	CCNB2		

4. Overview of networks inferred for each pathway by DTNI, per chemical group.

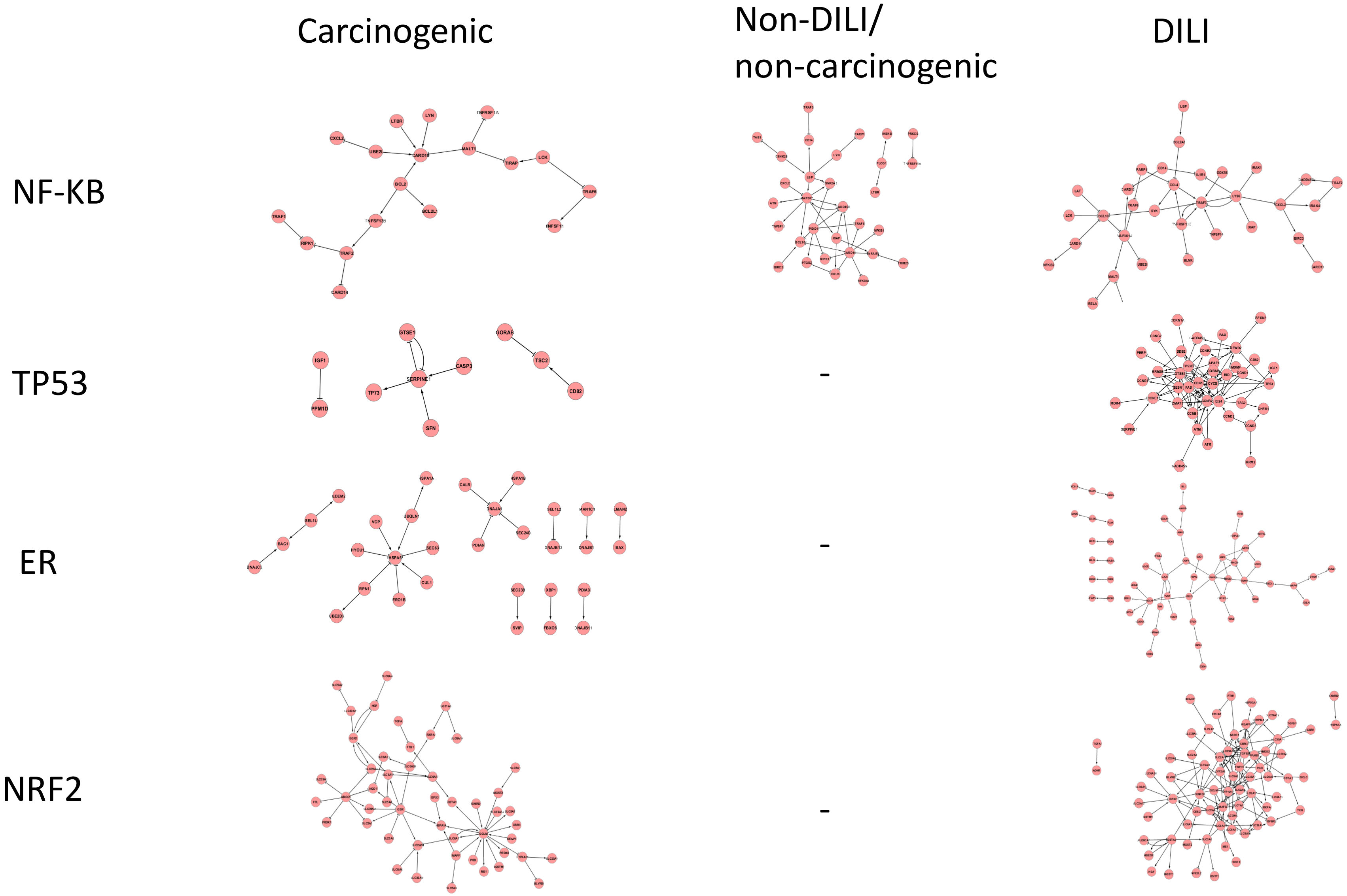

5. Validation of edges predicted by DTNI using annotated interactions available on CPDB.
    a. NRF2

Interaction<br>Source (interaction) target	Group	Type CPDB	Intermediary node	Is the intermediary node in the expression matrix?	Conclusion
ABCC5 (-1) FTL	Carcinogenic	-	-	-	novel
ABCC5 (-1) NQO1	Carcinogenic	-	-	-	novel
ABCC5 (-1) PRDX1	Carcinogenic	-	-	-	novel
ABCC5 (-1) SLC2A1	Carcinogenic	-	-	-	novel
ABCC5 (-1) SLC39A3	Carcinogenic	-	-	-	novel
ABCC5 (-1) SLC39A6	Carcinogenic	-	-	-	novel
ABCC5 (1) SLC39A2	Carcinogenic	-	-	-	novel
CYP4A11 (-1) BLVRB	Carcinogenic	-	-	-	novel
CYP4A11 (1) SLC39A4	Carcinogenic	-	-	-	novel
EGR1 (-1) HGF	Carcinogenic	-	-	-	novel
EGR1 (1) SLC39A3	Carcinogenic	-	-	-	novel
GCLM (-1) CYP4A11	Carcinogenic	-	-	-	novel
GCLM (1) CBR3	Carcinogenic	-	-	-	novel
GCLM (1) GSTA1	Carcinogenic	-	-	-	novel
GCLM (1) HSPA1A	Carcinogenic	-	-	-	novel
GCLM (1) ME1	Carcinogenic	-	-	-	novel
GCLM (1) PRDX6	Carcinogenic	-	-	-	novel
GCLM (1) SLC5A7	Carcinogenic	-	-	-	novel
GCLM (1) SLC6A7	Carcinogenic	-	-	-	novel
GPX3 (1) HSPA1A	Carcinogenic	-	-	-	novel
GSR (1) HSPA1A	Carcinogenic	-	-	-	novel
GSR (1) NQO1	Carcinogenic	-	-	-	novel
GSR (1) SLC2A1	Carcinogenic	-	-	-	novel
GSR (1) SLC2A10	Carcinogenic	-	-	-	novel
GSR (1) SLC6A11	Carcinogenic	-	-	-	novel

Interaction<br>Source (interaction) target	Group	Type CPDB	Intermediary node	Is the intermediary node in the expression matrix?	Conclusion
HGF (1) EGR1	Carcinogenic	-	-	-	novel
KEAP1 (1) GCLM	Carcinogenic	-	NFE2L2	yes	indirect
MAFF (-1) SLC5A6	Carcinogenic	-	-	-	novel
MAFF (1) GCLM	Carcinogenic	-	-	-	novel
MAFF (1) HSPA1A	Carcinogenic	-	-	-	novel
MGST2 (-1) GCLM	Carcinogenic	-	-	-	novel
PGD (1) GCLM	Carcinogenic	-	-	-	novel
SLC39A11 (-1) SLC5A2	Carcinogenic	-	-	-	novel
SLC39A11 (1) EGR1	Carcinogenic	-	-	-	novel
SLC39A14 (-1) GCLM	Carcinogenic	-	-	-	novel
SLC39A2 (-1) GSR	Carcinogenic	-	-	-	novel
SLC39A3 (1) EGR1	Carcinogenic	-	-	-	novel
SLC39A3 (1) SLC6A17	Carcinogenic	-	-	-	novel
SLC39A8 (1) SLC2A10	Carcinogenic	-	-	-	novel
SLC5A1 (1) MGST2	Carcinogenic	-	-	-	novel
SLC5A5 (-1) GSR	Carcinogenic	-	-	-	novel
SLC6A11 (-1) SLC5A8	Carcinogenic	-	-	-	novel
SLC6A11 (1) EGR1	Carcinogenic	-	-	-	novel
SLC6A11 (1) SLC6A17	Carcinogenic	-	-	-	novel
SLC6A15 (-1) NQO1	Carcinogenic	-	-	-	novel
SLC6A17 (1) FTH1	Carcinogenic	-	-	-	novel
SLC6A17 (1) GSTA1	Carcinogenic	-	-	-	novel
SLC6A20 (-1) GSR	Carcinogenic	-	-	-	novel
SLC6A20 (1) RXRA	Carcinogenic	-	-	-	novel
SLC6A20 (1) SLC6A15	Carcinogenic	-	-	-	novel
SLC6A4 (-1) HGF	Carcinogenic	-	-	-	novel
SLC6A6 (-1) SLC2A10	Carcinogenic	-	-	-	novel

Interaction<br>Source (interaction) target	Group	Type CPDB	Intermediary node	Is the intermediary node in the expression matrix?	Conclusion
SLC6A7 (-1) SLC2A10	Carcinogenic	-	-	-	novel
SLC6A7 (1) GCLM	Carcinogenic	-	-	-	novel
SQSTM1 (1) GCLM	Carcinogenic	-	-	-	novel
TGFA (-1) FTH1	Carcinogenic	-	-	-	novel
TXNRD1 (1) GCLM	Carcinogenic	-	-	-	novel
UGT1A6 (1) RXRA	Carcinogenic	-	-	-	novel
UGT1A6 (1) SLC6A14	Carcinogenic	-	-	-	novel
ABCC5 (-1) SLC2A1	DILI	-	-	-	novel
ALDH3A1 (-1) GSTA3	DILI	-	-	-	novel
CBR3 (-1) SERPINA1	DILI	-	-	-	novel
CBR3 (-1) SLC39A10	DILI	-	-	-	novel
CBR3 (1) HSP90AA1	DILI	-	-	-	novel
CBR3 (1) SLC2A1	DILI	-	-	-	novel
CBR3 (1) SLC39A12	DILI	-	-	-	novel
CBR3 (1) SLC5A8	DILI	-	-	-	novel
CES2 (-1) BLVRB	DILI	-	-	-	novel
CES2 (-1) MAFG	DILI	-	-	-	novel
CES2 (-1) SLC7A11	DILI	-	-	-	novel
CES2 (1) GPX2	DILI	-	-	-	novel
CES2 (1) MGST2	DILI	-	-	-	novel
CYP2A6 (-1) ABCC5	DILI	-	-	-	novel
CYP2A6 (-1) MAFG	DILI	-	-	-	novel
CYP2A6 (-1) SLC39A10	DILI	-	-	-	novel
CYP2A6 (1) CBR3	DILI	-	-	-	novel
CYP2A6 (1) SLC2A1	DILI	-	-	-	novel
CYP4A11 (1) HMOX1	DILI	-	-	-	novel
CYP4A11 (1) SLC2A1	DILI	-	-	-	novel

Interaction<br>Source (interaction) target	Group	Type CPDB	Intermediary node	Is the intermediary node in the expression matrix?	Conclusion
EPHA2 (1) SLC39A10	DILI	-	-	-	novel
FGF13 (-1) CBR3	DILI	-	-	-	novel
FGF13 (1) ABCC5	DILI	-	-	-	novel
FGF13 (1) SLC2A1	DILI	-	-	-	novel
FGF13 (1) SLC7A11	DILI	-	-	-	novel
FTH1 (-1) ABCC5	DILI	-	-	-	novel
FTH1 (-1) EPHA2	DILI	-	-	-	novel
GCLC (1) SLC6A9	DILI	-	-	-	novel
GCLM (-1) CYP4A11	DILI	-	-	-	novel
GCLM (-1) GPX2	DILI	-	-	-	novel
GCLM (1) BLVRB	DILI	-	-	-	novel
GCLM (1) MAFG	DILI	-	-	-	novel
GCLM (1) SLC6A16	DILI	-	-	-	novel
GCLM (1) SLC6A9	DILI	-	-	-	novel
GPX2 (-1) SLC6A19	DILI	-	-	-	novel
GSTA1 (1) TXN	DILI	-	-	-	novel
GSTA3 (-1) ALDH3A1	DILI	-	-	-	novel
GSTA3 (1) HBEGF	DILI	-	-	-	novel
GSTA3 (1) HGF	DILI	-	-	-	novel
GSTA3 (1) MGST3	DILI	biochemical reaction	-	-	direct
GSTA3 (1) SLC5A1	DILI	-	-	-	novel
GSTA3 (1) TXNRD3	DILI	-	-	-	novel
GSTM5 (1) GPX2	DILI	-	-	-	novel
HBEGF (1) GSTA3	DILI	-	-	-	novel
HMOX1 (-1) TGFB1	DILI	-	-	-	novel
HMOX1 (1) SLC6A9	DILI	-	-	-	novel
ME1 (1) MAFG	DILI	-	-	-	novel

Interaction<br>Source (interaction) target	Group	Type CPDB	Intermediary node	Is the intermediary node in the expression matrix?	Conclusion
PGD (-1) ABCC5	DILI	-	-	-	novel
PGD (-1) CBR3	DILI	-	-	-	novel
PGD (-1) SLC2A6	DILI	-	-	-	novel
PGD (-1) SLC39A4	DILI	-	-	-	novel
PGD (1) FGF13	DILI	-	-	-	novel
PGD (1) RXRA	DILI	-	-	-	novel
PPARD (1) GSTA1	DILI	-	-	-	novel
PPARD (1) KEAP1	DILI	-	-	-	novel
PPARD (1) SLC2A1	DILI	-	-	-	novel
PPARD (1) SLC6A9	DILI	-	-	-	novel
SERPINA1 (-1) CBR3	DILI	-	-	-	novel
SERPINA1 (1) ABCC5	DILI	-	-	-	novel
SLC2A1 (-1) PPARD	DILI	-	-	-	novel
SLC2A1 (-1) TXNRD3	DILI	-	-	-	novel
SLC2A1 (1) CYP4A11	DILI	-	-	-	novel
SLC2A12 (-1) GPX2	DILI	-	-	-	novel
SLC2A2 (-1) DNAJB1	DILI	-	-	-	novel
SLC2A4 (1) SLC2A2	DILI	-	-	-	novel
SLC2A4 (1) TXNRD3	DILI	-	-	-	novel
SLC2A8 (-1) BLVRB	DILI	-	-	-	novel
SLC2A8 (-1) SLC2A6	DILI	biochemical reaction	-	-	direct
SLC2A8 (-1) SLC39A7	DILI	-	-	-	novel
SLC2A8 (1) CES2	DILI	-	-	-	novel
SLC2A8 (1) CYP4A11	DILI	-	-	-	novel
SLC2A8 (1) GPX2	DILI	-	-	-	novel
SLC2A9 (-1) SLC5A10	DILI	biochemical reaction	-	-	direct
SLC2A9 (-1) SLC6A16	DILI	-	-	-	novel

Interaction<br>Source (interaction) target	Group	Type CPDB	Intermediary node	Is the intermediary node in the expression matrix?	Conclusion
SLC2A9 (1) GPX2	DILI	-	-	-	novel
SLC2A9 (1) SLC2A6	DILI	biochemical reaction	-	-	direct
SLC39A1 (1) CYP4A11	DILI	-	-	-	novel
SLC39A1 (1) SLC39A10	DILI	biochemical reaction	-	-	direct
SLC39A14 (-1) CBR1	DILI	-	-	-	novel
SLC39A14 (-1) CBR3	DILI	-	-	-	novel
SLC39A14 (-1) PPARD	DILI	-	-	-	novel
SLC39A14 (-1) SLC6A9	DILI	-	-	-	novel
SLC39A14 (1) SERPINA1	DILI	-	-	-	novel
SLC39A6 (-1) SLC2A1	DILI	-	-	-	novel
SLC5A1 (-1) CYP4A11	DILI	-	-	-	novel
SLC5A1 (-1) NFE2L2	DILI	-	-	-	novel
SLC5A1 (1) GSTP1	DILI	-	-	-	novel
SLC5A1 (1) SLC39A3	DILI	-	-	-	novel
SLC5A10 (1) RXRA	DILI	-	-	-	novel
SLC5A10 (1) SLC39A11	DILI	-	-	-	novel
SLC5A10 (1) SLC39A3	DILI	-	-	-	novel
SLC5A10 (1) TGFBR2	DILI	-	-	-	novel
SLC5A8 (-1) MAFG	DILI	-	-	-	novel
SLC5A8 (-1) SLC2A6	DILI	-	-	-	novel
SLC5A8 (-1) SLC39A10	DILI	-	-	-	novel
SLC5A8 (-1) SLC7A11	DILI	-	-	-	novel
SLC5A8 (1) CBR3	DILI	-	-	-	novel
SLC5A8 (1) PPARD	DILI	-	-	-	novel
SLC5A8 (1) SLC2A1	DILI	-	-	-	novel
SLC5A8 (1) SLC39A3	DILI	-	-	-	novel
SLC6A11 (-1) MAFG	DILI	-	-	-	novel

Interaction<br>Source (interaction) target	Group	Type CPDB	Intermediary node	Is the intermediary node in the expression matrix?	Conclusion
SLC6A11 (-1) SLC39A10	DILI	-	-	-	novel
SLC6A11 (-1) SLC39A11	DILI	-	-	-	novel
SLC6A11 (-1) SLC7A11	DILI	-	-	-	novel
SLC6A11 (1) FGF13	DILI	-	-	-	novel
SLC6A11 (1) RXRA	DILI	-	-	-	novel
SLC6A11 (1) SLC39A3	DILI	-	-	-	novel
SLC6A11 (1) TGFBR2	DILI	-	-	-	novel
SLC6A13 (-1) CYP4A11	DILI	-	-	-	novel
SLC6A13 (-1) SLC2A9	DILI	-	-	-	novel
SLC6A14 (1) GPX2	DILI	-	-	-	novel
SLC6A15 (-1) SLC2A6	DILI	-	-	-	novel
SLC6A16 (-1) CYP4A11	DILI	-	-	-	novel
SLC6A16 (-1) SLC2A9	DILI	-	-	-	novel
SLC6A16 (1) SLC39A11	DILI	-	-	-	novel
SLC6A16 (1) SLC6A19	DILI	-	-	-	novel
SLC6A16 (1) SOD3	DILI	-	-	-	novel
SLC6A16 (1) TXNRD3	DILI	-	-	-	novel
SLC6A20 (-1) GPX2	DILI	-	-	-	novel
SLC7A11 (-1) TGFBR2	DILI	-	-	-	novel
SLC7A11 (1) SLC6A13	DILI	-	-	-	novel
TGFA (1) ADH7	DILI	-	-	-	novel
TGFB2 (-1) KEAP1	DILI	-	-	-	novel
TGFB2 (-1) SLC2A1	DILI	-	-	-	novel
TGFB2 (1) ABCC5	DILI	-	-	-	novel
TGFB2 (1) SLC2A2	DILI	-	-	-	novel
TGFB2 (1) SLC2A6	DILI	-	-	-	novel
TGFB2 (1) SLC39A10	DILI	-	-	-	novel

Interaction<br>Source (interaction) target	Group	Type CPDB	Intermediary node	Is the intermediary node in the expression matrix?	Conclusion
TGFB2 (1) SLC39A4	DILI	-	-	-	novel
TXN (1) TGFBR2	DILI	-	-	-	novel
TXNRD1 (1) HSPA1A	DILI	-	-	-	novel
TXNRD3 (-1) CBR3	DILI	-	-	-	novel
TXNRD3 (1) MAFG	DILI	-	-	-	novel
CYP4A11 (-1) CBR1	Non-DILI/Non-carc.	-	-	-	novel
SOD3 (1) PTGR1	Non-DILI/Non-carc.	-	-	-	novel

b. NF-KB

Interaction<br>Source (interaction) target	Group	Type CPDB	Intermediary nodes	Is the intermediary node in the expression matrix?	Conclusion
BCL2 (1) TNFSF13B	Carcinogenic	-	BIRC2, BIRC3	yes	indirect
BCL2 (1) CARD10	Carcinogenic	-	-		novel
BCL2A1 (-1) CD14	Carcinogenic	-	-		novel
BCL2A1 (-1) CSNK2A1	Carcinogenic	-	-		novel
BCL2A1 (1) DDX58	Carcinogenic	-	-		novel
LCK (-1) TRAF6	Carcinogenic	-	IKBKG	no	direct
LCK (1) TIRAP	Carcinogenic	-	BTK, MAP3K7	yes	indirect
LTBR (1) CARD10	Carcinogenic	-	-		novel
LYN (1) CARD10	Carcinogenic	-	-		novel
TRAF1 (-1) RIPK1	Carcinogenic	-	TICAM1	yes	indirect
TRAF2 (-1) RIPK1	Carcinogenic	protein interaction and biochemical reaction	-		direct
TRAF2 (-1) CARD14	Carcinogenic	-	-		novel
TRAF6 (1) TNFSF11	Carcinogenic	-	-		novel
UBE2I (-1) CXCL2	Carcinogenic	-	-		novel
UBE2I (1) CARD10	Carcinogenic	-	-		novel
TNFSF13B (1) TRAF2	Carcinogenic	-	CD40, MAP3K14	yes	indirect
MALT1 (-1) TNFRSF1A	Carcinogenic	-	-		novel
MALT1 (-1) CARD10	Carcinogenic	-	-		novel
MALT1 (-1) TIRAP	Carcinogenic	-	MAP3K7, TRAF6	yes	indirect
BIRC2 (1) BCL10	Non-DILI/non-carcinogenic	protein interaction and biochemical reaction	-		direct
XIAP (1) CHUK	Non-DILI/non-carcinogenic	-	NFKB1, UBE2D1, UBE2D2		indirect
XIAP (1) GADD45B	Non-DILI/non-carcinogenic	-	-		novel

Interaction<br>Source (interaction) target	Group	Type CPDB	Intermediary nodes	Is the intermediary node in the expression matrix?	Conclusion
XIAP (1) TNFAIP3	Non-DILI/non-carcinogenic	-	-		novel
CSNK2B (1) LBP	Non-DILI/non-carcinogenic	-	-		novel
CSNK2B (-1) TAB1	Non-DILI/non-carcinogenic	-	-		novel
CXCL2 (-1) MAP3K7	Non-DILI/non-carcinogenic	-	-		novel
LBP (1) CD14	Non-DILI/non-carcinogenic	gene regulatory interaction, biochemical interaction and protein interaction	-		direct
LBP (1) CSNK2A2	Non-DILI/non-carcinogenic	-	-		novel
LBP (1) MAP3K7	Non-DILI/non-carcinogenic	-	-		novel
LYN (-1) PARP1	Non-DILI/non-carcinogenic	-	RELA, TRAF6	yes	indirect
LYN (-1) LBP	Non-DILI/non-carcinogenic	-	-		novel
GADD45B (1) MAP3K7	Non-DILI/non-carcinogenic	-	-		novel
PLCG1 (1) IKBKB	Non-DILI/non-carcinogenic	-	ATM	yes	indirect
PLCG1 (1) LTBR	Non-DILI/non-carcinogenic	-	-		novel
PRKCB (-1) TNFRSF11A	Non-DILI/non-carcinogenic	-	-		novel
PTGS2 (-1) CHUK	Non-DILI/non-carcinogenic	-	-		novel
MAP3K7 (1) XIAP	Non-DILI/non-carcinogenic	-	-		direct
MAP3K7 (1) ATM	Non-DILI/non-carcinogenic	-	-		novel

Interaction<br>Source (interaction) target	Group	Type CPDB	Intermediary nodes	Is the intermediary node in the expression matrix?	Conclusion
MAP3K7 (1) CSNK2A2	Non-DILI/non-carcinogenic	-	-		novel
MAP3K7 (1) GADD45B	Non-DILI/non-carcinogenic	-	-		novel
MAP3K7 (1) TNFSF11	Non-DILI/non-carcinogenic	-	-		novel
MAP3K7 (1) BCL10	Non-DILI/non-carcinogenic	gene regulatory interaction	-		direct
TRAF3 (-1) CD14	Non-DILI/non-carcinogenic	-	TLR3, TLR4	yes	indirect
TRIM25 (-1) TNFAIP3	Non-DILI/non-carcinogenic	-	DDX58	yes	indirect
PIDD1 (-1) CSNK2A2	Non-DILI/non-carcinogenic	-	-		novel
PIDD1 (-1) GADD45B	Non-DILI/non-carcinogenic	-	-		novel
PIDD1 (1) PTGS2	Non-DILI/non-carcinogenic	-	-		novel
PIDD1 (-1) TRAF6	Non-DILI/non-carcinogenic	-	-		novel
PIDD1 (-1) RIPK1	Non-DILI/non-carcinogenic	biochemical reaction	-		direct
PIDD1 (-1) BCL10	Non-DILI/non-carcinogenic	-	RIPK1		indirect
CARD14 (-1) CHUK	Non-DILI/non-carcinogenic	-	PRKCB		indirect
CARD14 (-1) GADD45B	Non-DILI/non-carcinogenic	-	-		novel
CARD14 (-1) NFKB1	Non-DILI/non-carcinogenic	-	-		novel
CARD14 (-1) NFKBIA	Non-DILI/non-carcinogenic	-	-		novel
CARD14 (-1) TNFAIP3	Non-DILI/non-carcinogenic	-	-		novel

Interaction<br>Source (interaction) target	Group	Type CPDB	Intermediary nodes	Is the intermediary node in the expression matrix?	Conclusion
CARD14 (-1) TRAF6	Non-DILI/non-carcinogenic	-	-		novel
CARD14 (-1) RIPK1	Non-DILI/non-carcinogenic	-	-		novel
CARD14 (-1) BCL10	Non-DILI/non-carcinogenic	-	-		novel
PARP1 (-1) CCL4	DILI	-	-		novel
PARP1 (-1) TRAF6	DILI	-	ATM		indirect
XIAP (1) LY96	DILI	-	-		novel
BCL2A1 (-1) CCL4	DILI	-	-		novel
CD14 (-1) IL1R1	DILI	-	-		novel
CD14 (-1) CARD10	DILI	-	-		novel
CXCL2 (1) BIRC3	DILI	-	-		novel
CXCL2 (-1) GADD45B	DILI	-	-		novel
CXCL2 (1) IRAK4	DILI	-	-		novel
LBP (1) BCL2A1	DILI	-	-		novel
LCK (-1) BCL10	DILI	-	IKBKG/MAP3K7		direct/indirect
CCL4 (1) SYK	DILI	-	-		novel
CCL4 (1) TRAF3	DILI	-	-		novel
CCL21 (-1) MALT1	DILI	-	-		novel
SYK (1) TRAF3	DILI	-	-		direct
SYK (-1) BCL10	DILI	-	NFKBIA		indirect
TRAF2 (1) GADD45B	DILI	-	-		novel
TRAF2 (-1) IRAK4	DILI	-	-		novel
TRAF3 (-1) LY96	DILI	-	TLR4		indirect
TRAF3 (1) TNFRSF13C	DILI	protein interaction	-		direct
TNFSF14 (1) TRAF3	DILI	protein interaction	-		direct
BCL10 (-1) MAP3K14	DILI	-	CHUK, IRAK1, BIRC2		indirect

Interaction<br>Source (interaction) target	Group	Type CPDB	Intermediary nodes	Is the intermediary node in the expression matrix?	Conclusion
MAP3K14 (-1) TRAF6	DILI	protein interaction	-		direct
MAP3K14 (-1) UBE2I	DILI	-	-		novel
MAP3K14 (1) MALT1	DILI	-	NFKBI1, IKBKB, BIRC2		indirect
MAP3K14 (1) CARD10	DILI	-	-		novel
MALT1 (-1) RELA	DILI	-	UBA1, UBE2D1, TNFAIP3		indirect
DDX58 (1) TRAF3	DILI	protein interaction	-		direct
LY96 (-1) CXCL2	DILI	-	-		novel
LY96 (-1) IL1R1	DILI	-	-		novel
LY96 (-1) IRAK1	DILI	-	-		novel
LY96 (-1) TRAF3	DILI	-	-		novel
LAT (-1) BCL10	DILI	-	TRAF6		indirect
CARD14 (1) NFKB2	DILI	-	-		novel
CARD14 (-1) BCL10	DILI	-	-		novel
CARD11 (1) BIRC3	DILI	-	-		novel
TNFRSF13C (1) CCL4	DILI	-	-		novel
TNFRSF13C (1) TRAF3	DILI	-	-		novel
TNFRSF13C (-1) BLNK	DILI	-	-		novel

c. ER

Interaction<br>Source (interaction) target	Group	Type CPDB	Intermediary node	Is the intermediary node in the expression matrix?	Conclusion
CALR (-1) DNAJA1	Carcinogenic	-	-	-	novel
CUL1 (1) HSPA6	Carcinogenic	-	-	-	direct
DNAJC3 (1) BAG1	Carcinogenic	-	HSPA8	yes	indirect
ERO1B (-1) HSPA6	Carcinogenic	-	-	-	novel
HSPA1B (1) DNAJA1	Carcinogenic	-	-	-	novel
HYOU1 (-1) HSPA6	Carcinogenic	-	DNAJB1	yes	indirect
LMAN2 (1) BAX	Carcinogenic	-	-	-	novel
MAN1C1 (1) DNAJB1	Carcinogenic	-	-	-	novel
PDIA3 (1) DNAJB11	Carcinogenic	-	HSPA8	yes	indirect
PDIA6 (-1) DNAJA1	Carcinogenic	-	-	-	novel
RPN1 (-1) HSPA6	Carcinogenic	-	HSPA5	yes	indirect
RPN1 (1) UBE2D3	Carcinogenic	-	-	-	novel
SEC23B (1) SVIP	Carcinogenic	-	VCP, UFD1L	yes	indirect
SEC24D (-1) DNAJA1	Carcinogenic	-	-	-	novel
SEC63 (-1) HSPA6	Carcinogenic	-	-	-	novel
SEL1L (1) BAG1	Carcinogenic	-	-	-	novel
SEL1L (1) EDEM2	Carcinogenic	-	-	-	novel
SEL1L2 (-1) DNAJB12	Carcinogenic	-	-	-	novel
UBQLN1 (1) HSPA1A	Carcinogenic	-	-	-	novel
UBQLN1 (1) HSPA6	Carcinogenic	-	-	-	novel
VCP (1) HSPA6	Carcinogenic	-	CUL1	yes	indirect
XBP1 (1) FBXO6	Carcinogenic	-	-	-	novel
BAG1 (1) HSPA2	DILI	-	-	-	novel
BAX (-1) CALR	DILI	-	-	-	novel
BAX (-1) HSP90AB1	DILI	-	-	-	novel

Interaction<br>Source (interaction) target	Group	Type CPDB	Intermediary node	Is the intermediary node in the expression matrix?	Conclusion
CALR (-1) NFE2L2	DILI	-	-	-	novel
CALR (-1) YOD1	DILI	-	-	-	novel
CKAP4 (-1) CALR	DILI	-	-	-	novel
DNAJA1 (-1) CKAP4	DILI	-	-	-	novel
DNAJA1 (-1) PPP1R15A	DILI	-	HSPA8	yes	indirect
DNAJA1 (-1) UBE2D1	DILI	-	-	-	novel
DNAJA1 (-1) XBP1	DILI	-	-	-	novel
DNAJA1 (1) PRKCSH	DILI	-	-	-	novel
DNAJA1 (1) UBE2D2	DILI	-	-	-	novel
DNAJB12 (1) SEL1L	DILI	-	-	-	novel
ERLEC1 (1) RAD23A	DILI	-	-	-	novel
ERLEC1 (1) SEC61A1	DILI	-	-	-	novel
ERO1B (1) DDIT3	DILI	-	-	-	novel
FBXO2 (-1) PPP1R15A	DILI	-	-	-	novel
HERPUD1 (-1) PRKCSH	DILI	-	-	-	novel
HERPUD1 (1) P4HB	DILI	-	-	-	novel
HSP90AA1 (1) DNAJB1	DILI	-	-	-	novel
HSP90AB1 (-1) PARK2	DILI	-	-	-	novel
HSPA2 (1) UBE2D2	DILI	-	-	-	novel
HSPA8 (-1) EDEM1	DILI	-	-	-	novel
LMAN2 (-1) UBQLN1	DILI	-	-	-	novel
LMAN2 (1) CKAP4	DILI	-	-	-	novel
MAN1B1 (1) LMAN2	DILI	-	-	-	novel
MAN1B1 (1) SIL1	DILI	-	-	-	novel
MAPK8 (1) HSP90AA1	DILI	-	BCL2	yes	indirect
MAPK8 (1) SEC13	DILI	-	-	-	novel
MAPK8 (1) UBQLN3	DILI	-	-	-	novel

Interaction<br>Source (interaction) target	Group	Type CPDB	Intermediary node	Is the intermediary node in the expression matrix?	Conclusion
MBTPS2 (-1) SAR1B	DILI	-	-	-	novel
NGLY1 (-1) UBE4B	DILI	-	-	-	novel
NGLY1 (1) DERL2	DILI	-	-	-	novel
NGLY1 (1) SEC24C	DILI	-	-	-	novel
NGLY1 (1) SELENOS	DILI	-	-	-	novel
NGLY1 (1) UBE2D2	DILI	-	-	-	novel
PREB (1) EDEM2	DILI	-	-	-	novel
PRKCSH (-1) XBP1	DILI	-	-	-	novel
PRKCSH (1) SAR1B	DILI	-	-	-	novel
SAR1B (1) UFD1L	DILI	-	-	-	novel
SEC24D (1) EIF2AK3	DILI	-	-	-	novel
SEC31B (1) GANAB	DILI	-	-	-	novel
SEC31B (1) PLAA	DILI	-	-	-	novel
STUB1 (1) HSPA8	DILI	-	-	-	novel
STUB1 (1) UBE2D2	DILI	-	-	-	novel
TRAM1 (-1) DNAJA1	DILI	-	-	-	novel
TRAM1 (-1) PRKCSH	DILI	-	-	-	novel
TRAM1 (-1) SEC13	DILI	-	-	-	novel
TRAM1 (-1) SEC62	DILI	-	-	-	novel
TRAM1 (-1) UFD1L	DILI	-	-	-	novel
UBE2D1 (1) PPP1R15A	DILI	-	-	-	novel
UBE2D1 (1) XBP1	DILI	-	-	-	novel
UGGT1 (1) YOD1	DILI	-	-	-	novel
UGGT2 (1) CALR	DILI	-	-	-	novel
XBP1 (-1) SAR1B	DILI	-	-	-	novel
YOD1 (-1) CALR	DILI	-	-	-	novel

Interaction<br>Source (interaction) target	Group	Type CPDB	Intermediary node	Is the intermediary node in the expression matrix?	Conclusion
YOD1 (1) NGLY1	DILI	-	-	-	novel

d. TP53

Interaction<br>Source (interaction) target	Group	Type CPDB	Intermediary node	Is the intermediary in the expression matrix?	Conclusion
APAF1 (1) CDK1	DILI	biochemical reaction			direct
APAF1 (1) CCNB2	DILI		CDK1	yes	indirect
APAF1 (1) EI24	DILI				novel
APAF1 (1) GTSE1	DILI		TP53	yes	indirect
APAF1 (-1) CYCS	DILI				novel
APAF1 (-1) RFWD2	DILI		TP53	yes	indirect
FAS (1) CCNB1	DILI		GADD45A	yes	indirect
FAS (1) CCNE1	DILI		ATM	yes	indirect
FAS (1) CCNB2	DILI				novel
FAS (1) CCNE2	DILI		TP53	yes	indirect
FAS (1) EI24	DILI				novel
FAS (1) RRM2B	DILI		TP53, ATM	yes	indirect
FAS (1) CYCS	DILI		BID	yes	indirect
ATM (1) CCNB1	DILI		CDK1	yes	indirect
ATM (1) CDK1	DILI	biochemical reaction			direct
ATM (1) CCNB2	DILI				novel
ATM (1) EI24	DILI				novel
ATM (-1) GADD45G	DILI		ATF2	no	direct
ATM (1) CYCS	DILI		E2F1	no	direct
ATR (1) CCNB1	DILI		E2F1	no	indirect
ATR (1) CCNB2	DILI		CDK1, CDK2	yes	direct
BAX (-1) CYCS	DILI	biochemical reaction			direct
CCND1 (1) CCNB2	DILI		E2F3	no	direct
CCND1 (1) CYCS	DILI				novel
BID (-1) EI24	DILI				novel
BID (-1) GTSE1	DILI				novel

Interaction<br>Source (interaction) target	Group	Type CPDB	Intermediary node	Is the intermediary in the expression matrix?	Conclusion
BID (1) CYCS	DILI				direct
BID (1) RFWD2	DILI				novel
BID (1) GORAB	DILI				novel
CCNB1 (1) CDK1	DILI				novel
CCND2 (-1) CCND3	DILI	biochemical reaction			direct
CCND2 (1) EI24	DILI				novel
CCND3 (1) CHEK1	DILI		ATM, ATR, CDKN2A, CDK1	yes	indirect
CCND3 (1) RRM2	DILI		CDK1, CDK2		indirect
CDK1 (1) CCNB1	DILI	biochemical reaction			direct
CDK1 (1) CCNB2	DILI		E2F3	no	direct
CDK1 (-1) CYCS	DILI		E2F1/BCL2	no/yes	direct/indirect
CDKN1A (1) TP53I3	DILI		TP53	yes	indirect
DDB2 (1) CDK1	DILI		CDKN1A/CDK6/E2F 1	yes/yes/no	indirect/indirect /direct
DDB2 (1) RRM2B	DILI		TP53	yes	indirect
DDB2 (1) CYCS	DILI				novel
CD82 (1) EI24	DILI				novel
MDM2 (1) EI24	DILI				novel
MDM2 (-1) CYCS	DILI		E2F1/BCL2	no/yes	direct/indirect
MDM4 (-1) CCNE1	DILI		E2F1/CDKN1A/CDK 2,4,6/ATM	no/yes	direct/indirects
SERPINE1 (1) CCNE1	DILI				novel
TP53 (1) IGF1	DILI		IGFBP3	yes	indirect
TP53 (1) EI24	DILI				novel
TP53 (-1) CYCS	DILI		E2F1/BAX, BID, BCL2	no/yes	direct/indirects
TP53 (-1) RFWD2	DILI	gene regulatory interaction			direct

Interaction<br>Source (interaction) target	Group	Type CPDB	Intermediary node	Is the intermediary in the expression matrix?	Conclusion
TP53 (-1) GORAB	DILI		MDM2	yes	indirect
TSC2 (1) CHEK1	DILI		CDK1	yes	indirect
TSC2 (-1) IGF1	DILI				novel
TSC2 (-1) EI24	DILI				novel
CCNE2 (1) RFWD2	DILI				novel
EI24 (1) FAS	DILI				novel
EI24 (1) CCNB1	DILI				novel
TP53I3 (-1) CCNE1	DILI				novel
TP53I3 (1) CCNB2	DILI				novel
TP53I3 (1) EI24	DILI				novel
TP53I3 (1) RRM2B	DILI				novel
TP53I3 (-1) CYCS	DILI				novel
SESN1 (1) CCNB1	DILI				novel
SESN1 (-1) CCNE1	DILI				novel
SESN1 (1) CCNG1	DILI				novel
SESN1 (1) CDK1	DILI				novel
SESN1 (-1) GADD45B	DILI				novel
SESN1 (1) CCNB2	DILI				novel
SESN1 (1) RRM2B	DILI				novel
SESN1 (-1) CYCS	DILI				novel
SESN1 (-1) GORAB	DILI				novel
GTSE1 (1) CCNB1	DILI		E2F1/CDK1, CDK2		direct/indirects
GTSE1 (-1) CCNE1	DILI		E2F1/CDK1, CDK2		direct/indirects
GTSE1 (-1) CCNG1	DILI		TP53, CDK2		indirect
GTSE1 (-1) CCNG2	DILI				novel
GTSE1 (1) CDK1	DILI				direct
GTSE1 (1) CCNB2	DILI				novel

Interaction<br>Source (interaction) target	Group	Type CPDB	Intermediary node	Is the intermediary in the expression matrix?	Conclusion
GTSE1 (1) CCNE2	DILI		TP53		indirect
GTSE1 (1) EI24	DILI				novel
GTSE1 (1) RRM2B	DILI		TP53		indirect
GTSE1 (1) CYCS	DILI		CDK2		indirect
GTSE1 (-1) PERP	DILI		TP53		indirect
GTSE1 (1) ZMAT3	DILI				novel
RFWD2 (1) GADD45B	DILI		E2F1	no	direct
RFWD2 (1) CCNE2	DILI		TP53	yes	indirect
RFWD2 (1) CYCS	DILI		E2F1	no	direct
RFWD2 (-1) SESN2	DILI				novel
ZMAT3 (1) CCNB1	DILI				novel
ZMAT3 (-1) CCNE1	DILI				novel
ZMAT3 (1) CDK1	DILI				novel
ZMAT3 (1) CCNB2	DILI				novel
ZMAT3 (1) EI24	DILI				novel
ZMAT3 (1) RRM2B	DILI				novel
ZMAT3 (-1) CYCS	DILI				novel
CASP3 (1) SERPINE1	Carcinogenic		TP73, TP53	yes	indirect
SFN (1) SERPINE1	Carcinogenic		TP53	yes	indirect
IGF1 (-1) PPM1D	Carcinogenic		E2F1	yes	direct
CD82 (1) TSC2	Carcinogenic				novel
SERPINE1 (1) TP73	Carcinogenic				novel
SERPINE1 (-1) GTSE1	Carcinogenic				novel
GTSE1 (-1) SERPINE1	Carcinogenic		E2F1	no	direct
GORAB (-1) TSC2	Carcinogenic				novel

6. Investigation from novel interactions in NRF2 pathway: STRING results

a. CBR3

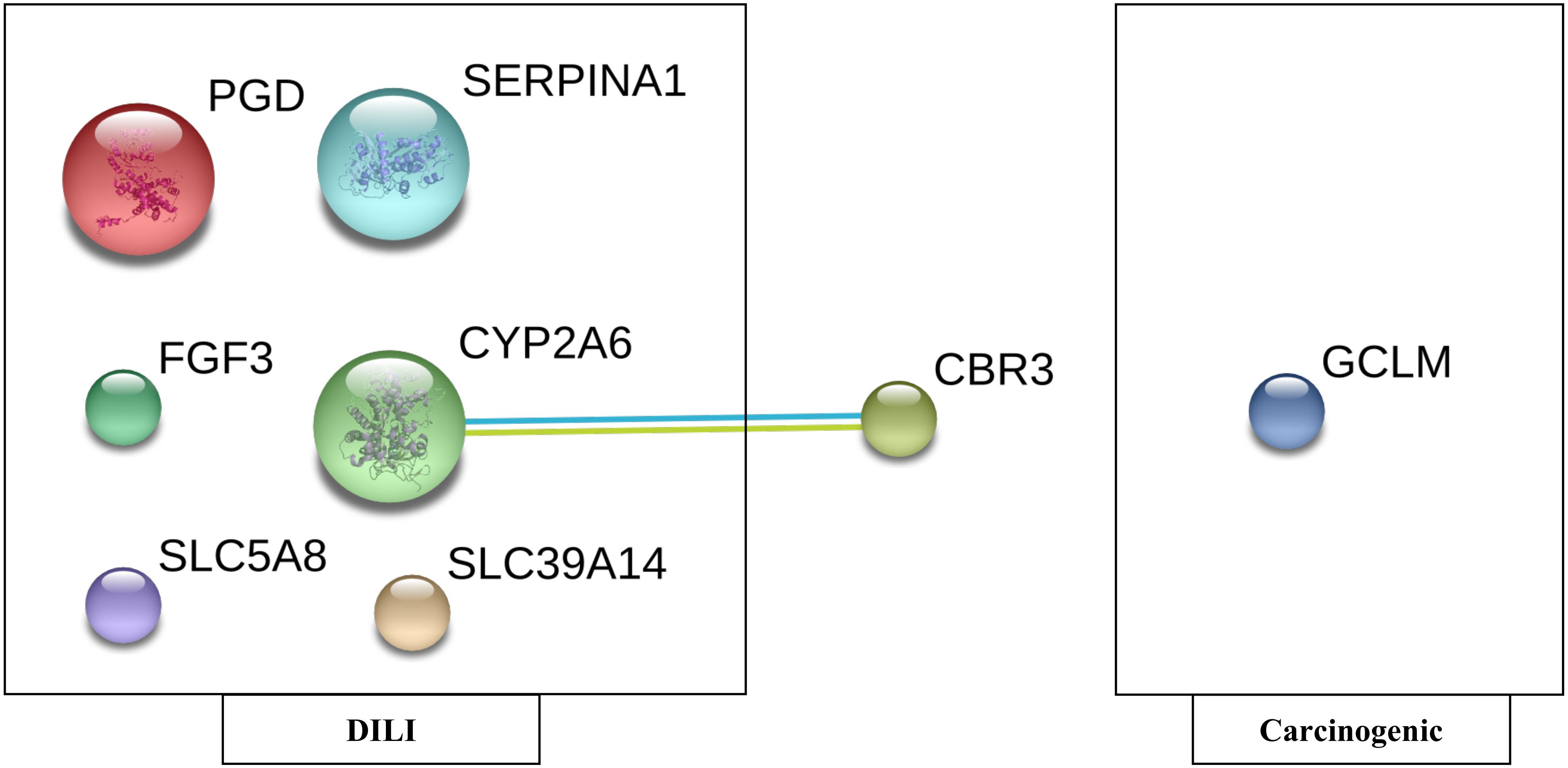

b. HGF

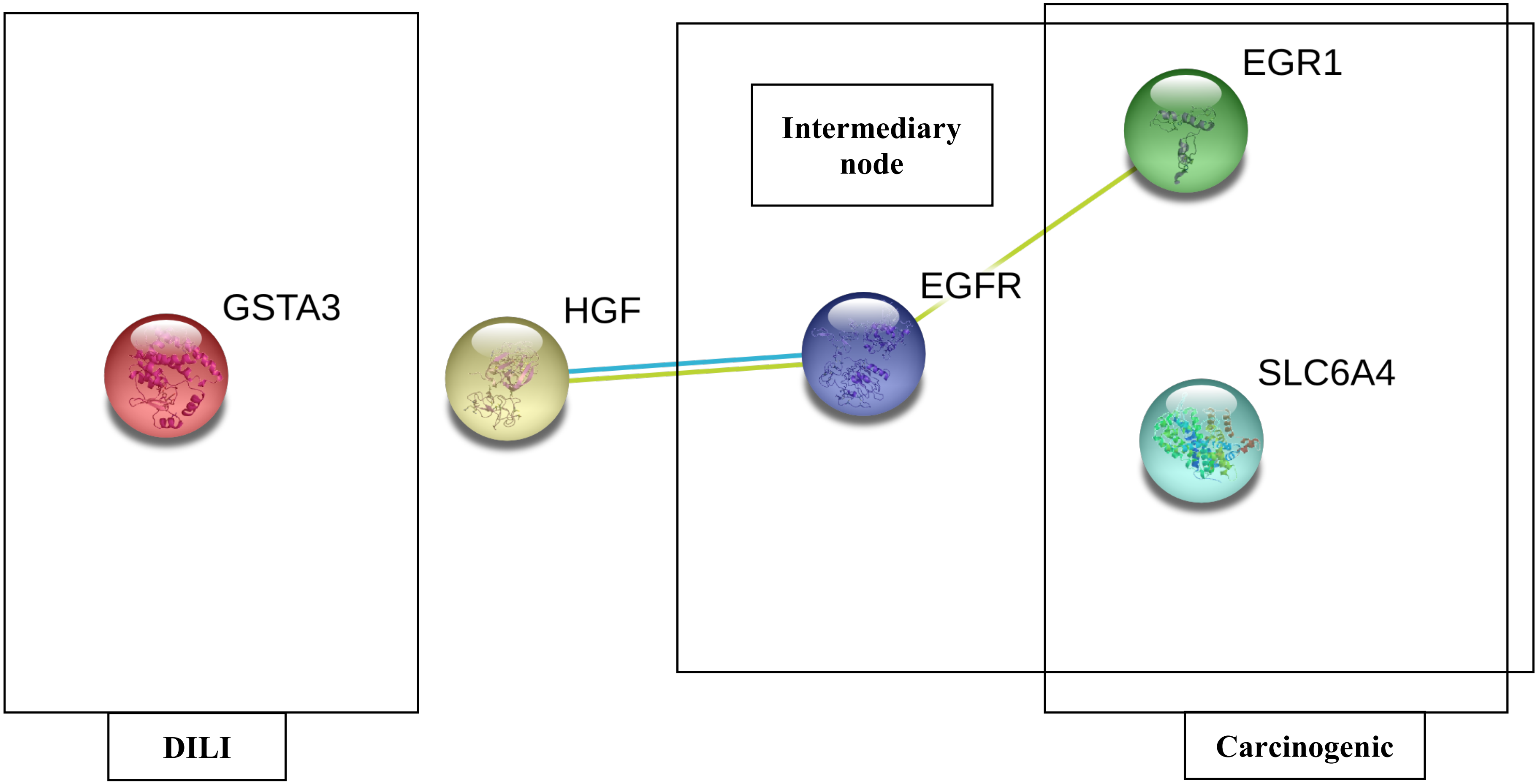
GSTA3
HGF
Intermediary node
EGFR
EGR1
SLC6A4
DILI
Carcinogenic

c. BLVRB

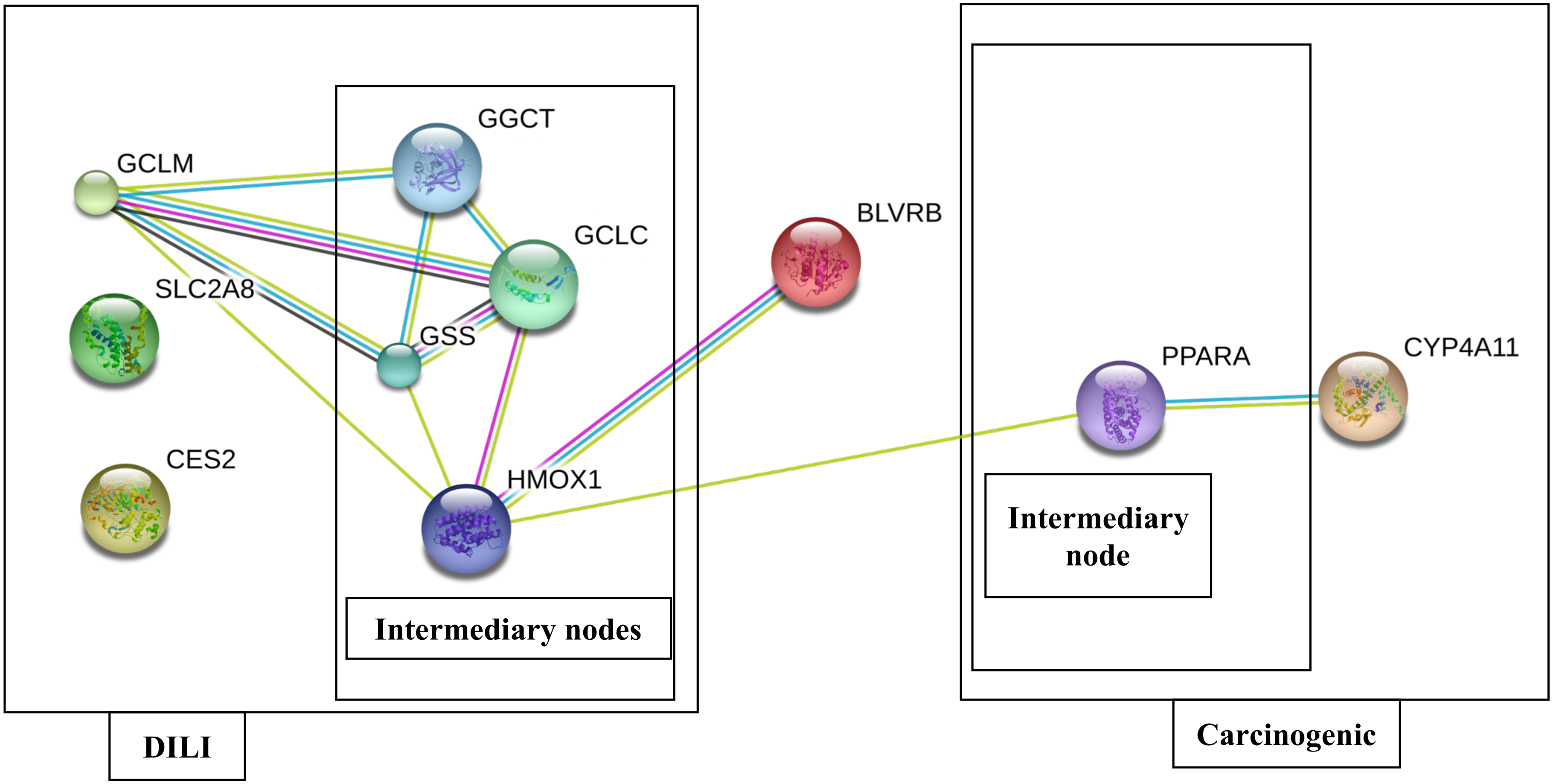
GCLM
GGCT
GCLC
SLC2A8
GSS
CES2
HMOX1
Intermediary nodes
DILI
BLVRB
PPARA
CYP4A11
Intermediary node
Carcinogenic
