# Supplementary material for: Dose and Time Dependencies in Stress Pathway Responses during Chemical Exposure: Novel Insights from Gene Regulatory Networks
Source: Front Genet. 2017 Oct 6;8:142. doi: 10.3389/fgene.2017.00142 (PMC5649202; doi:10.3389/fgene.2017.00142)
Supplement: Supplementary file 2 [file Presentation_1.PPTX]

## Slide 1
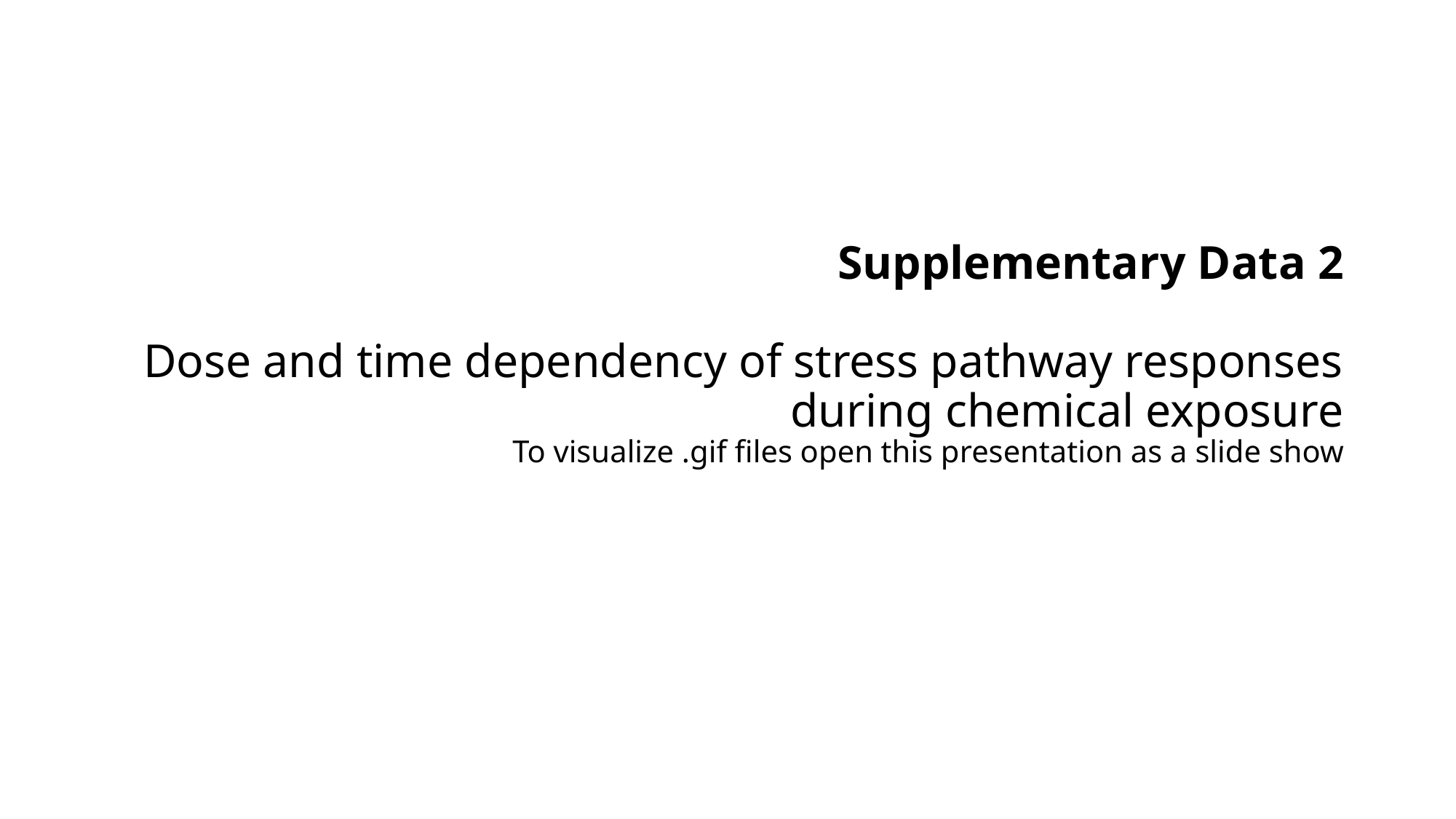

# Supplementary Data 2 Dose and time dependency of stress pathway responses during chemical exposure To visualize .gif files open this presentation as a slide show

## Slide 2
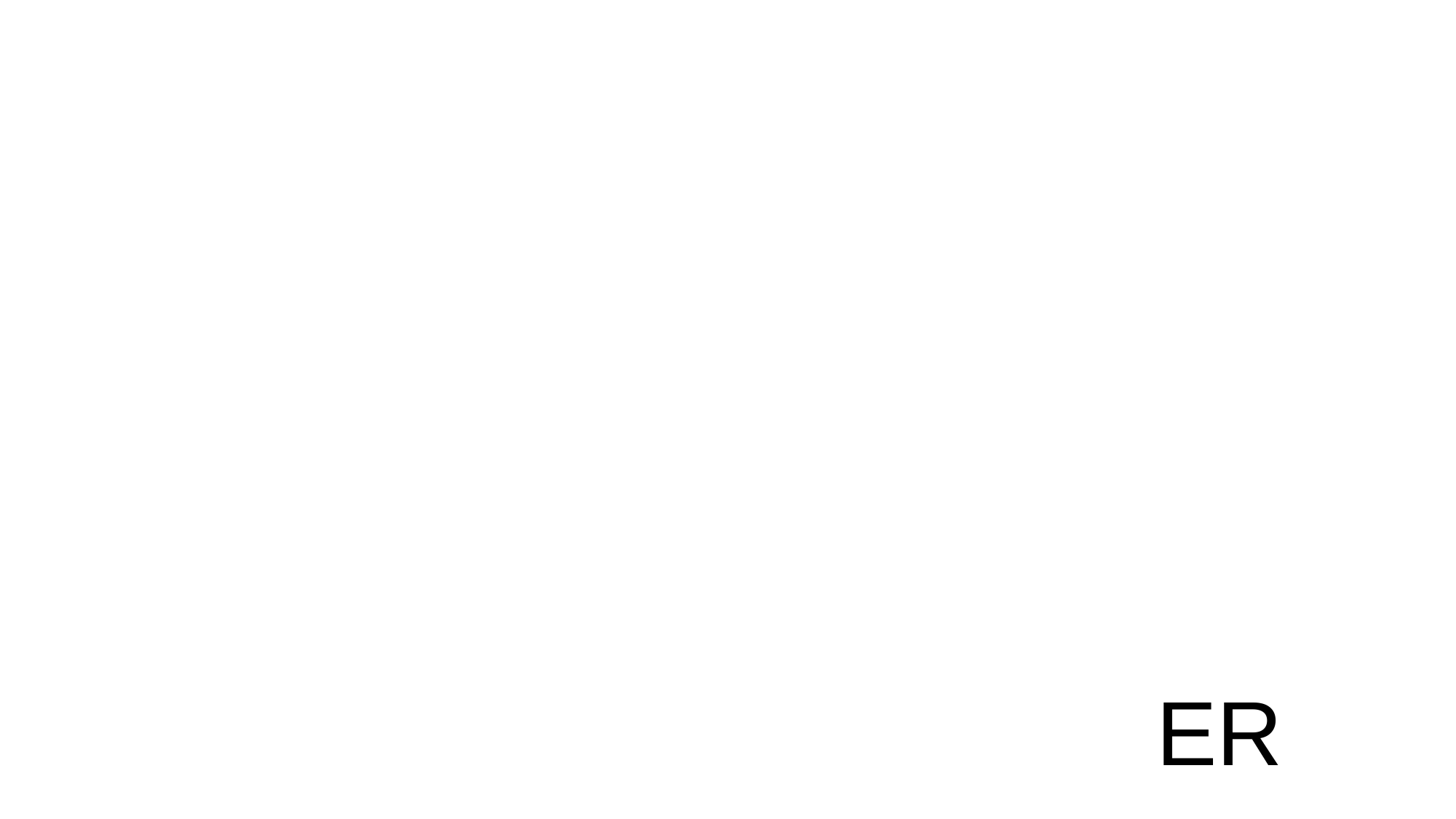

ER

## Slide 3
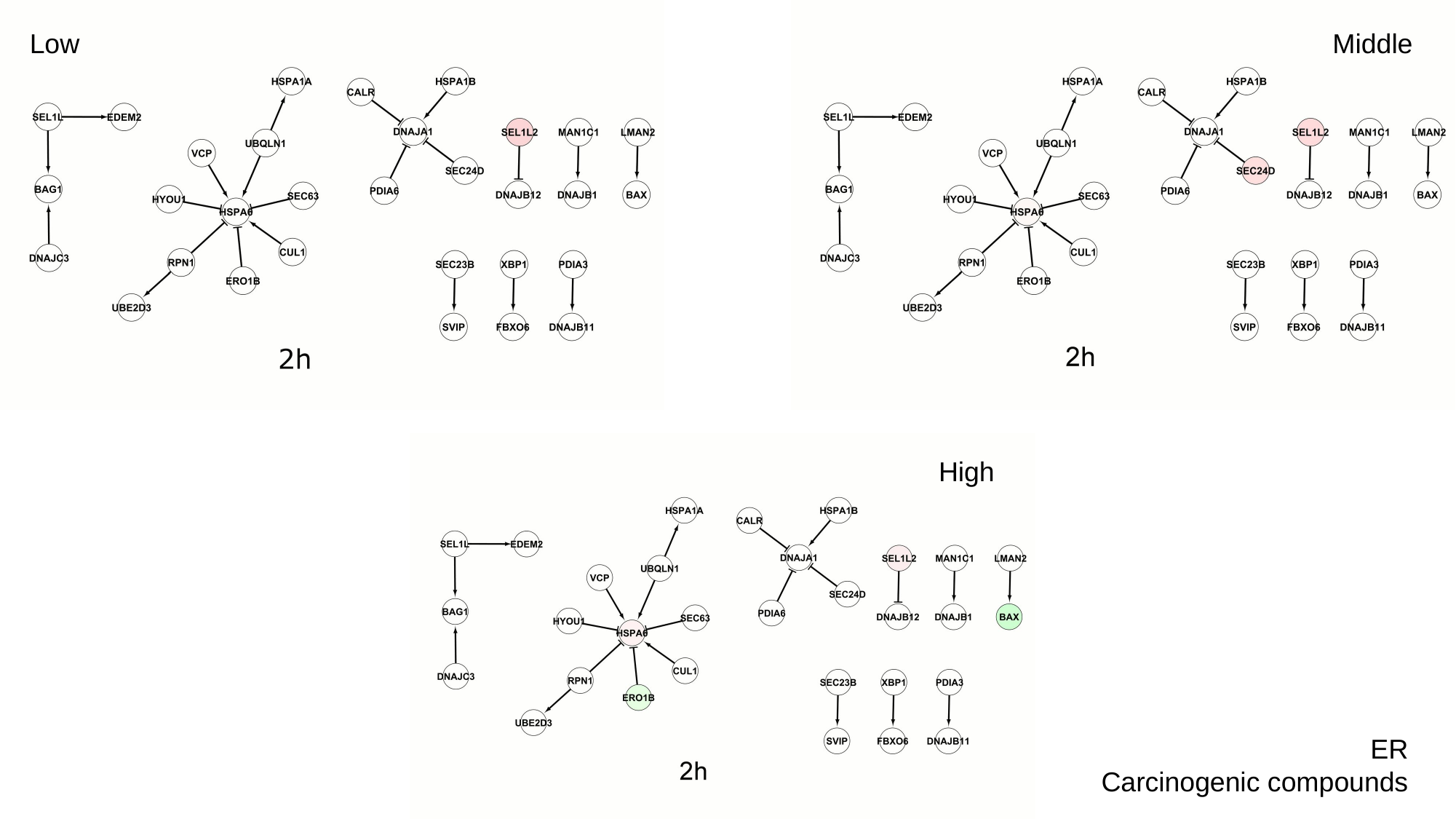

Low
Middle
High
ER
Carcinogenic compounds

## Slide 4
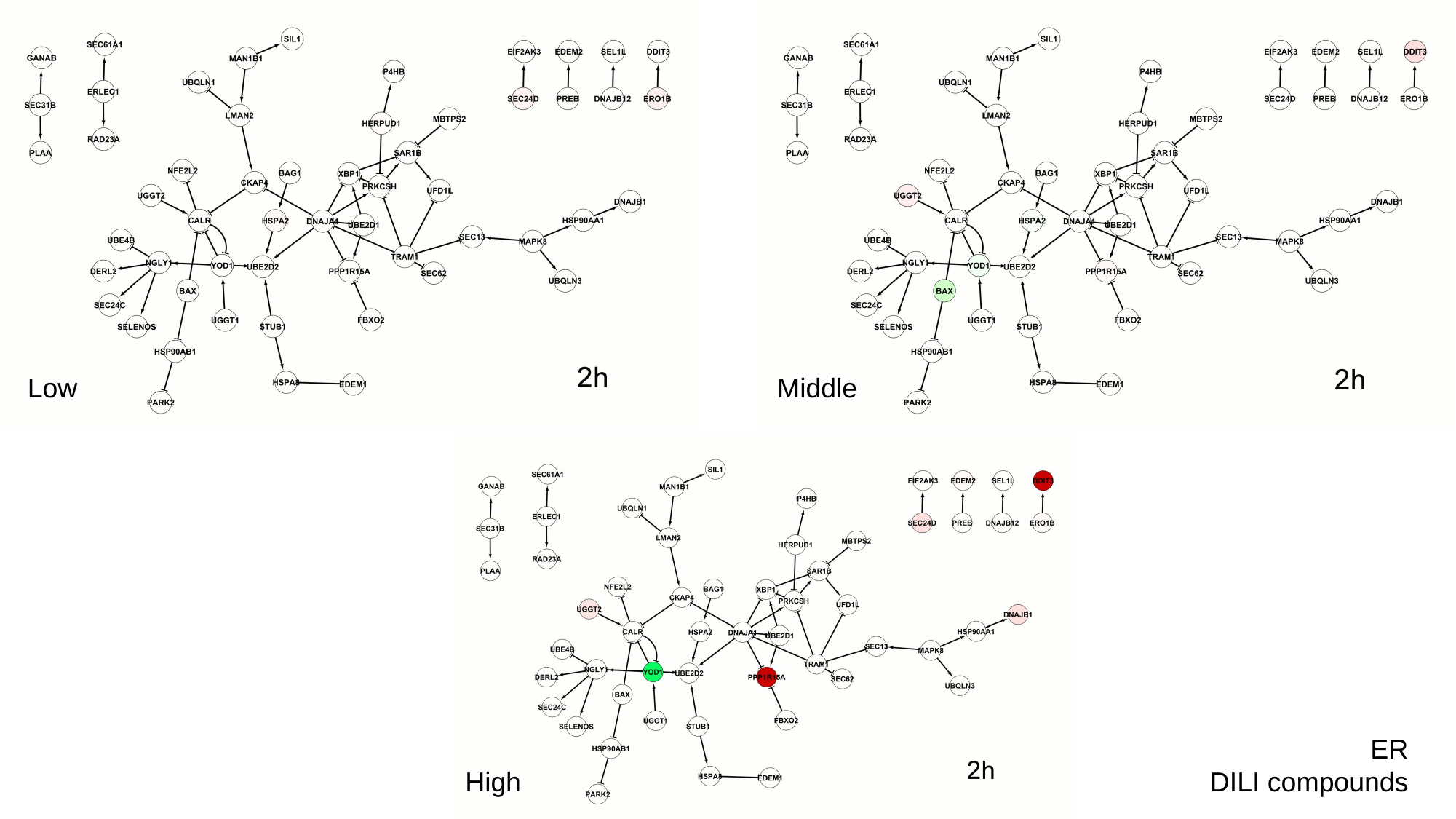

Low
Middle
ER
DILI compounds
High

## Slide 5
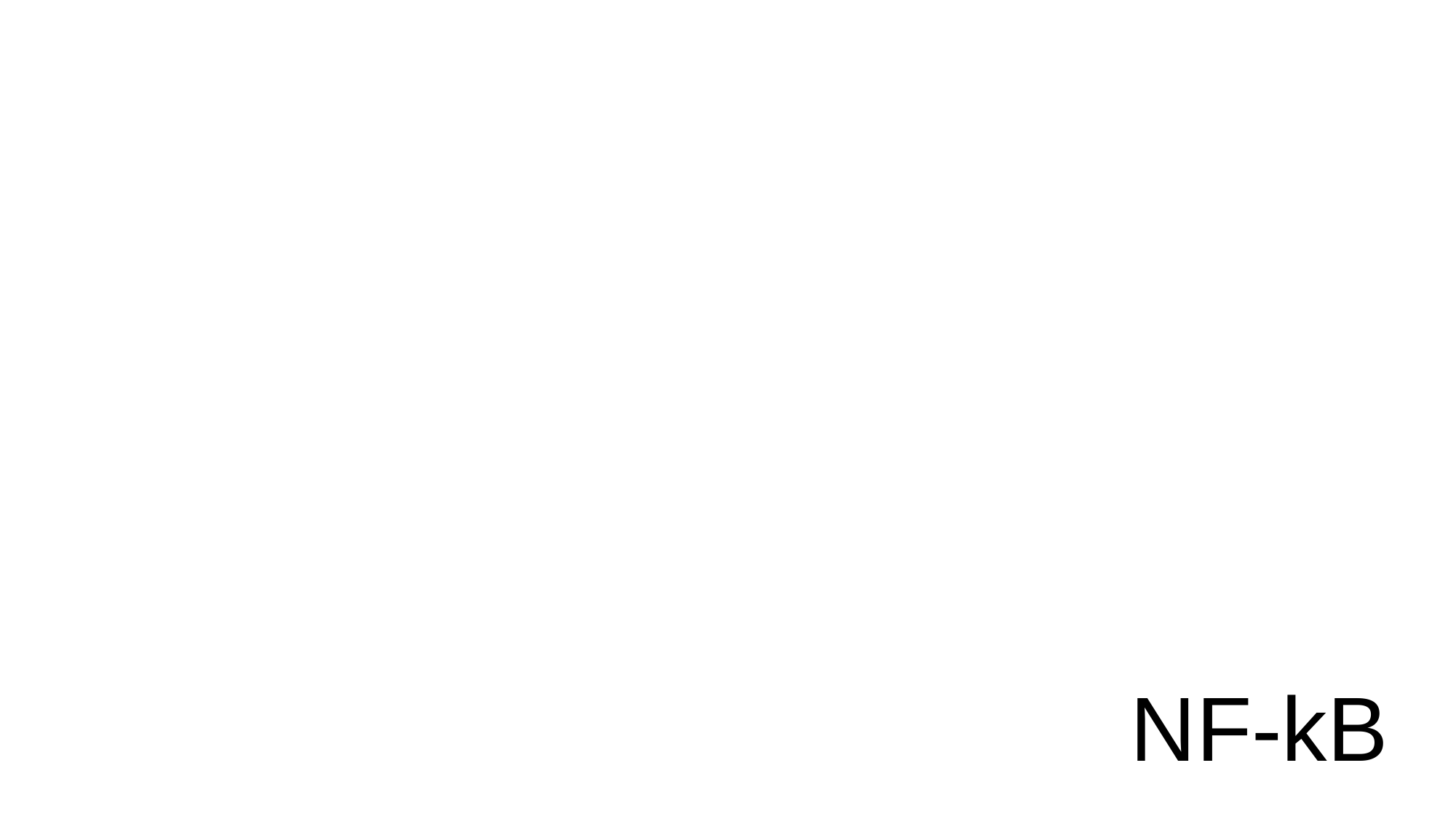

NF-kB

## Slide 6
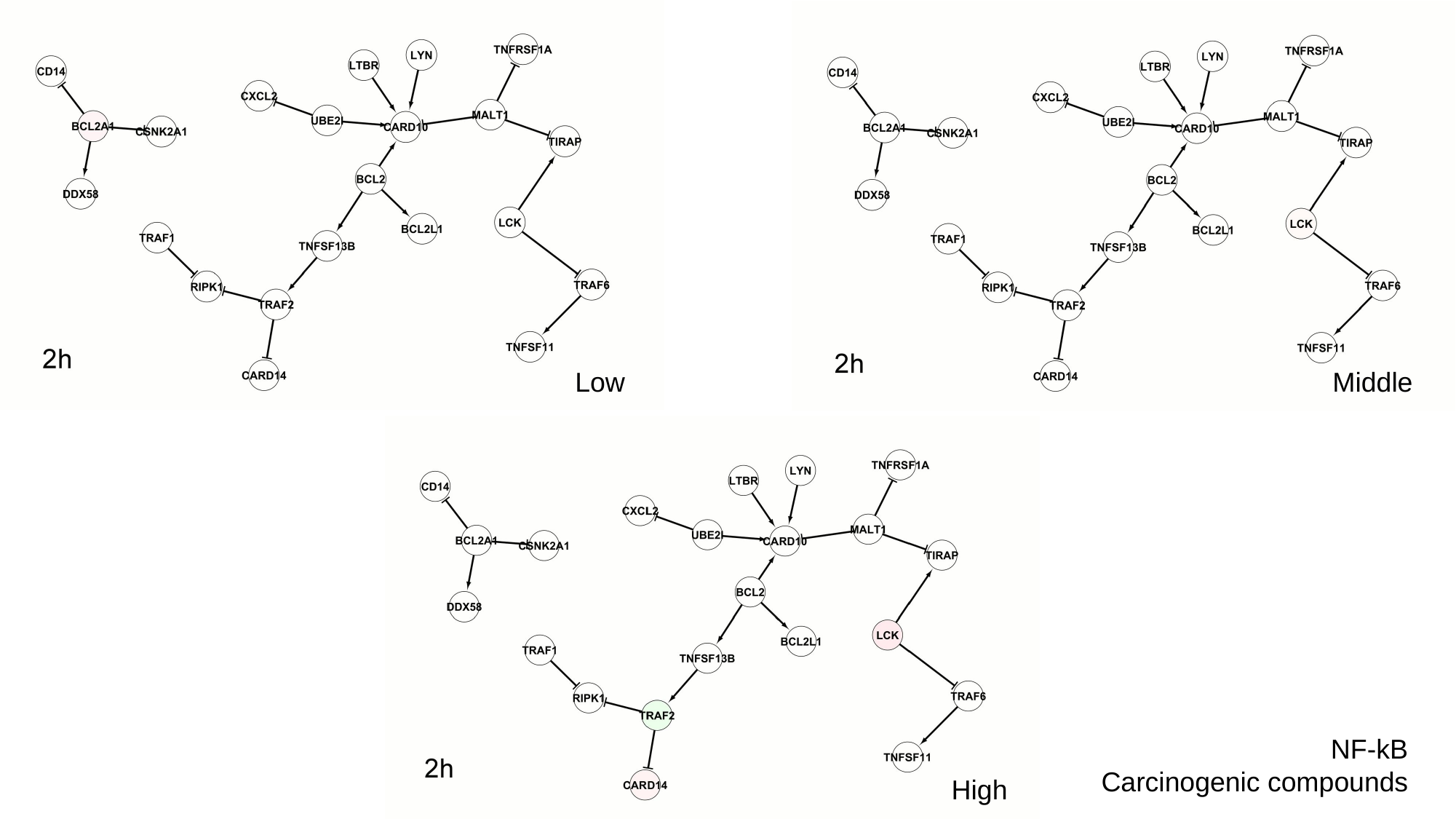

Low
Middle
NF-kB
Carcinogenic compounds
High

## Slide 7
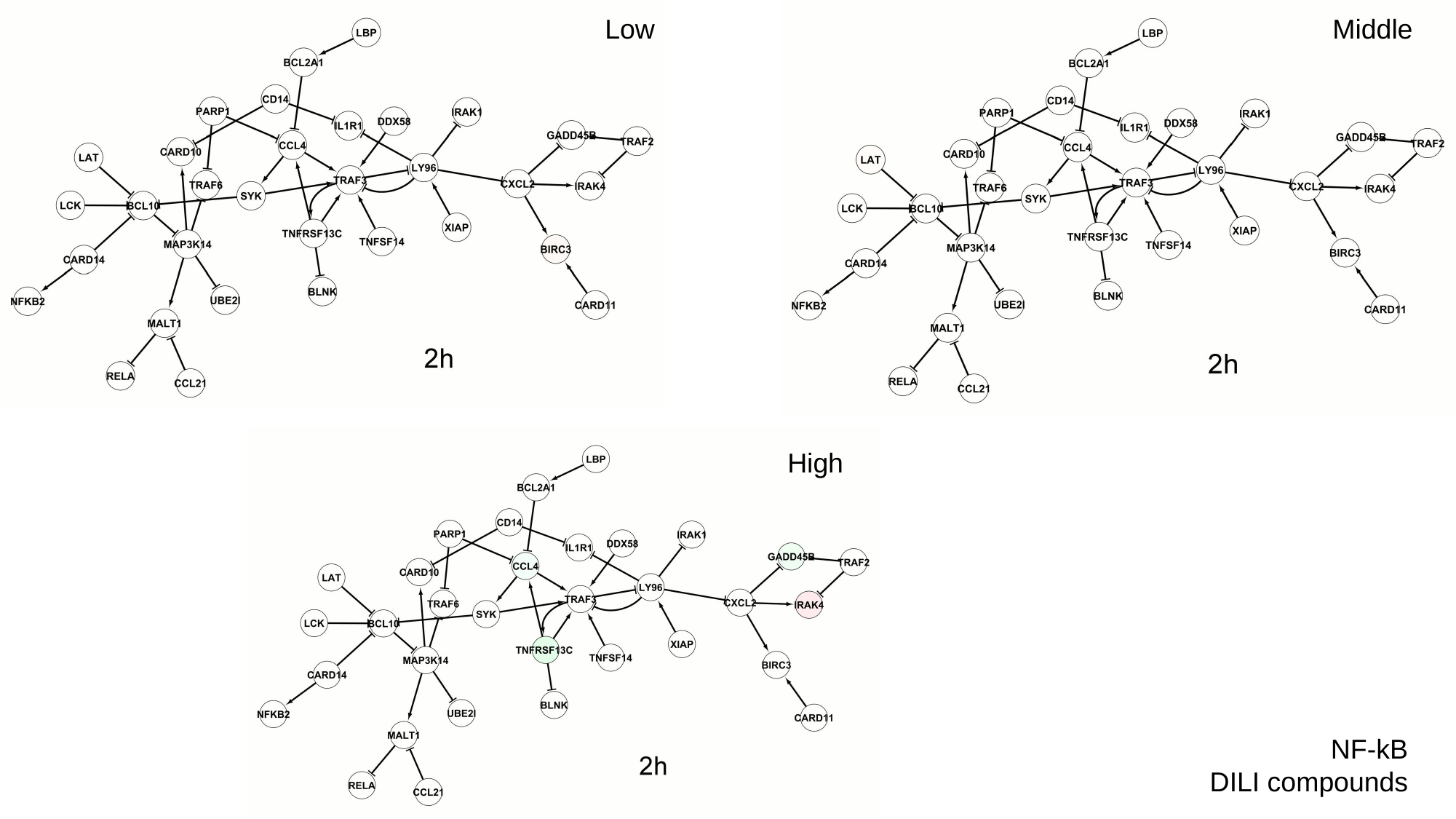

Low
Middle
High
NF-kB
DILI compounds

## Slide 8
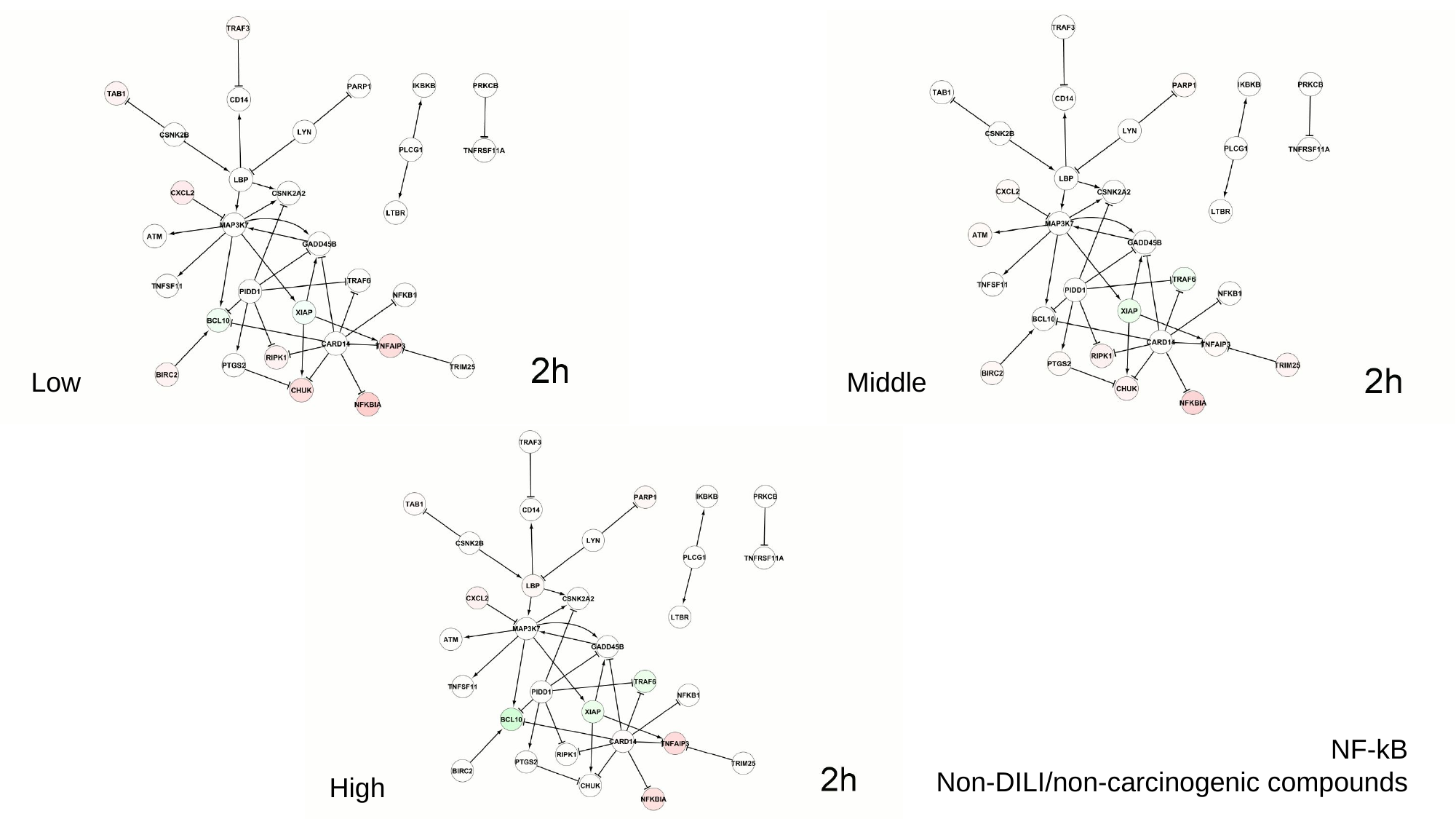

Low
Middle
NF-kB
Non-DILI/non-carcinogenic compounds
High

## Slide 9
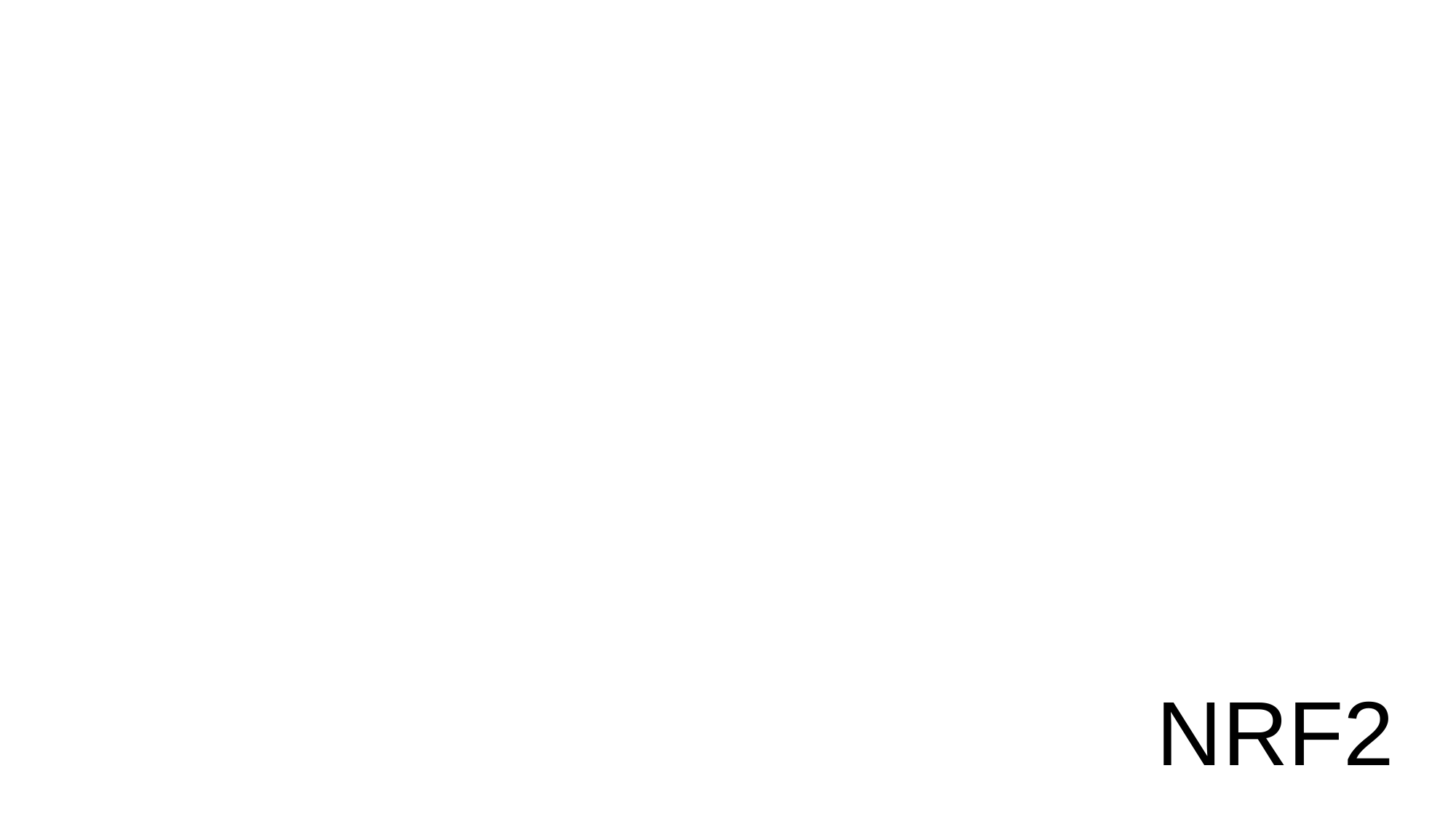

NRF2

## Slide 10
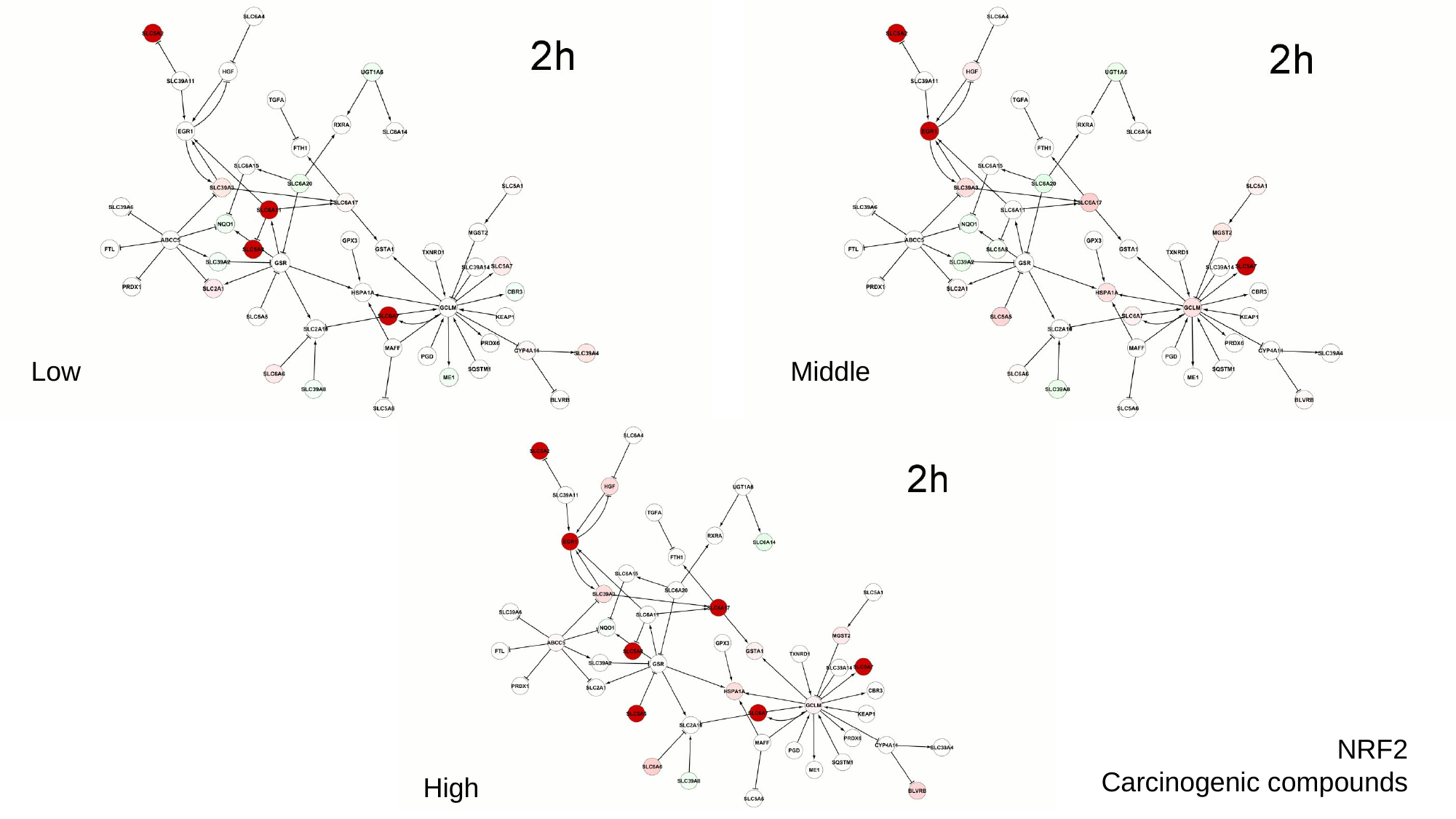

Low
Middle
NRF2
Carcinogenic compounds
High

## Slide 11
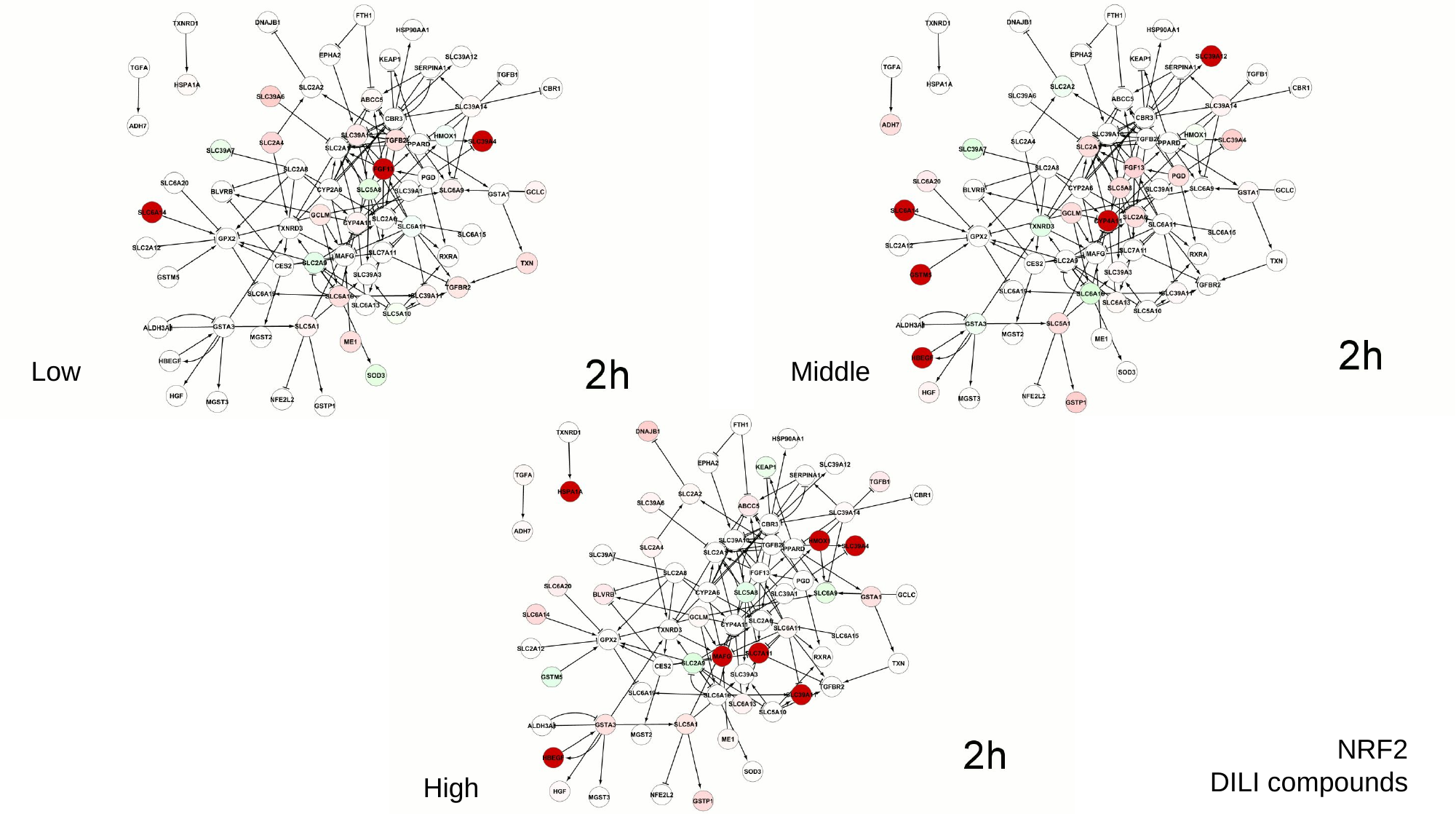

Low
Middle
NRF2
DILI compounds
High

## Slide 12
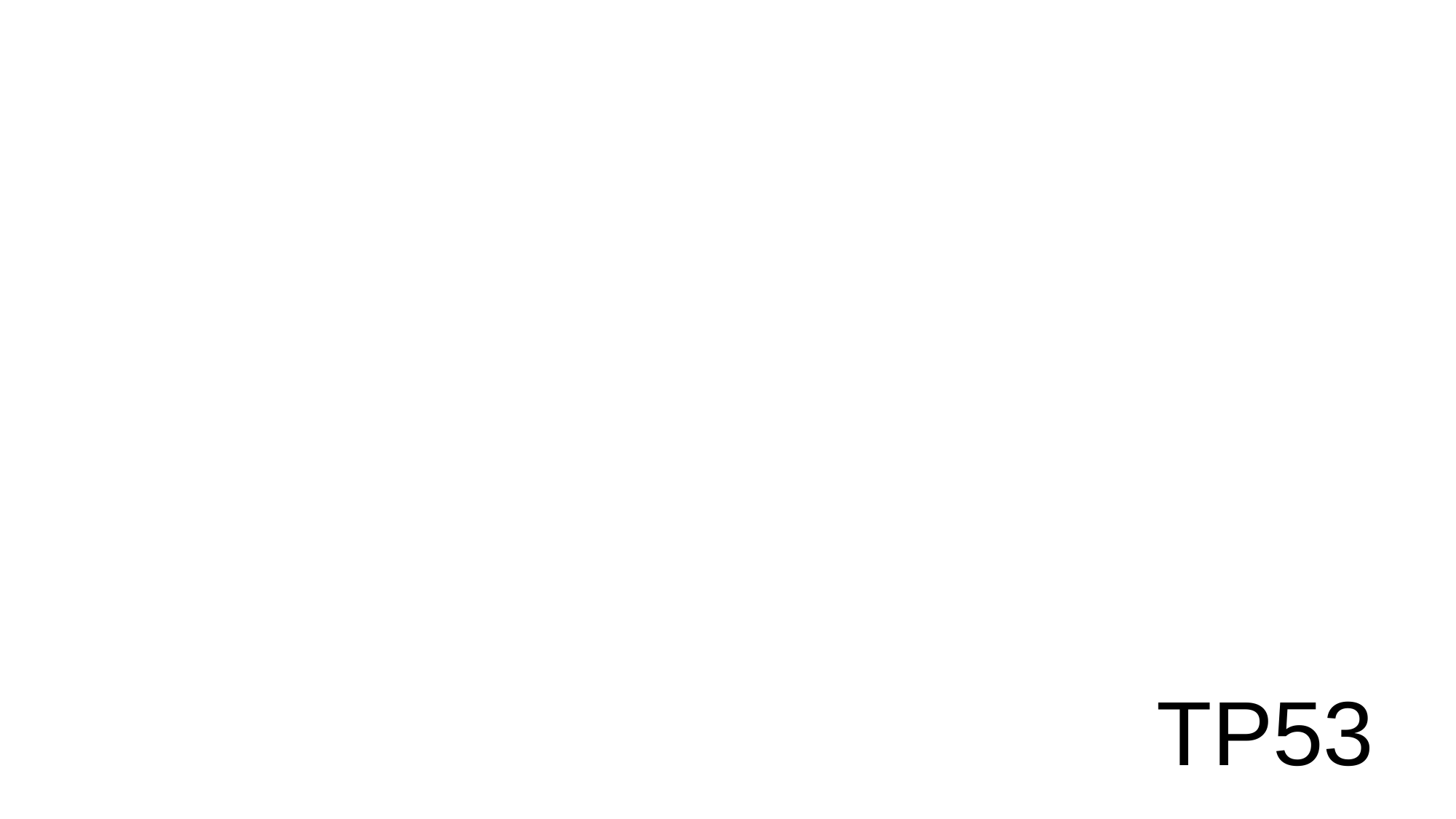

TP53

## Slide 13
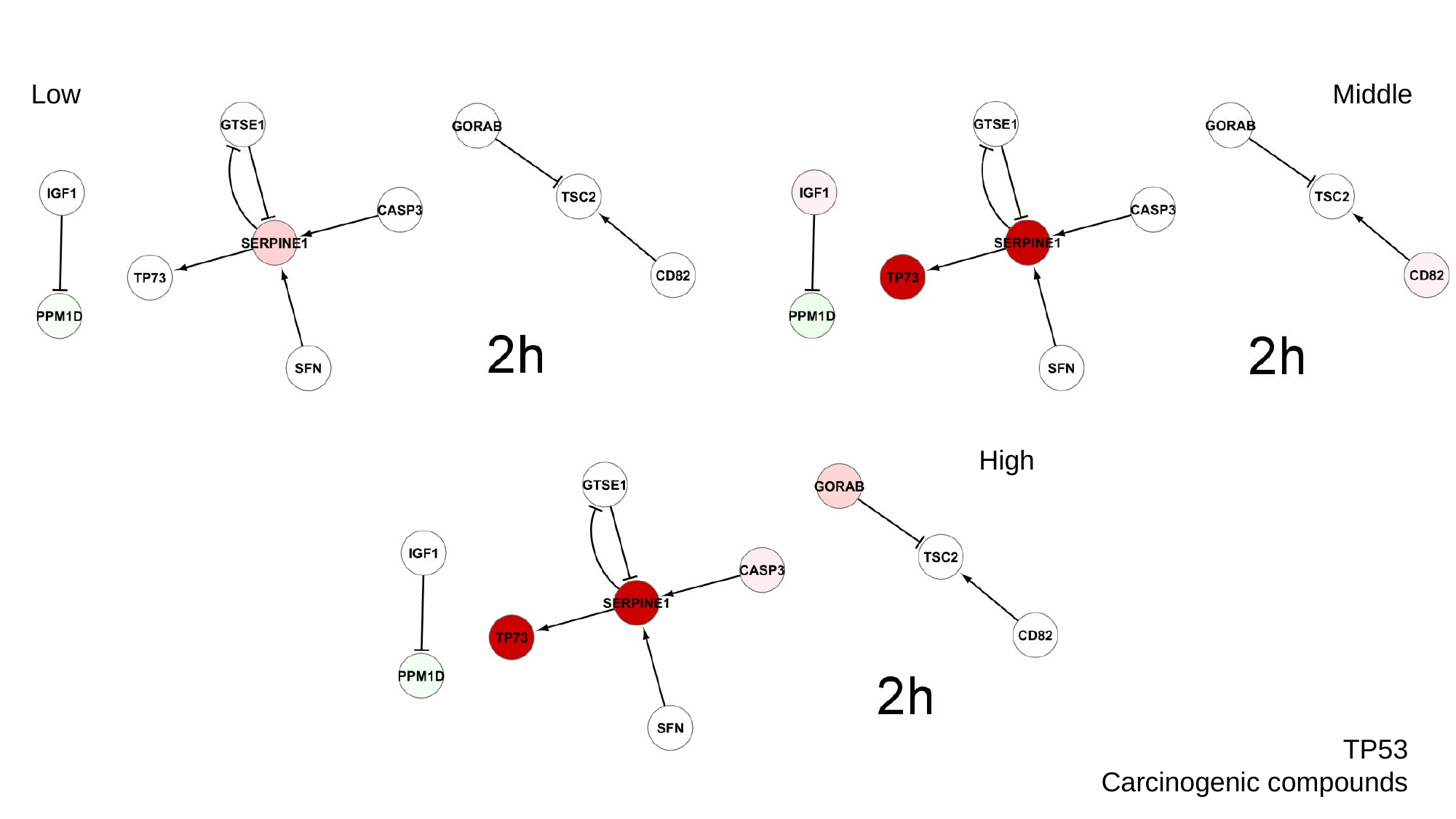

Low
Middle
High
TP53
Carcinogenic compounds

## Slide 14
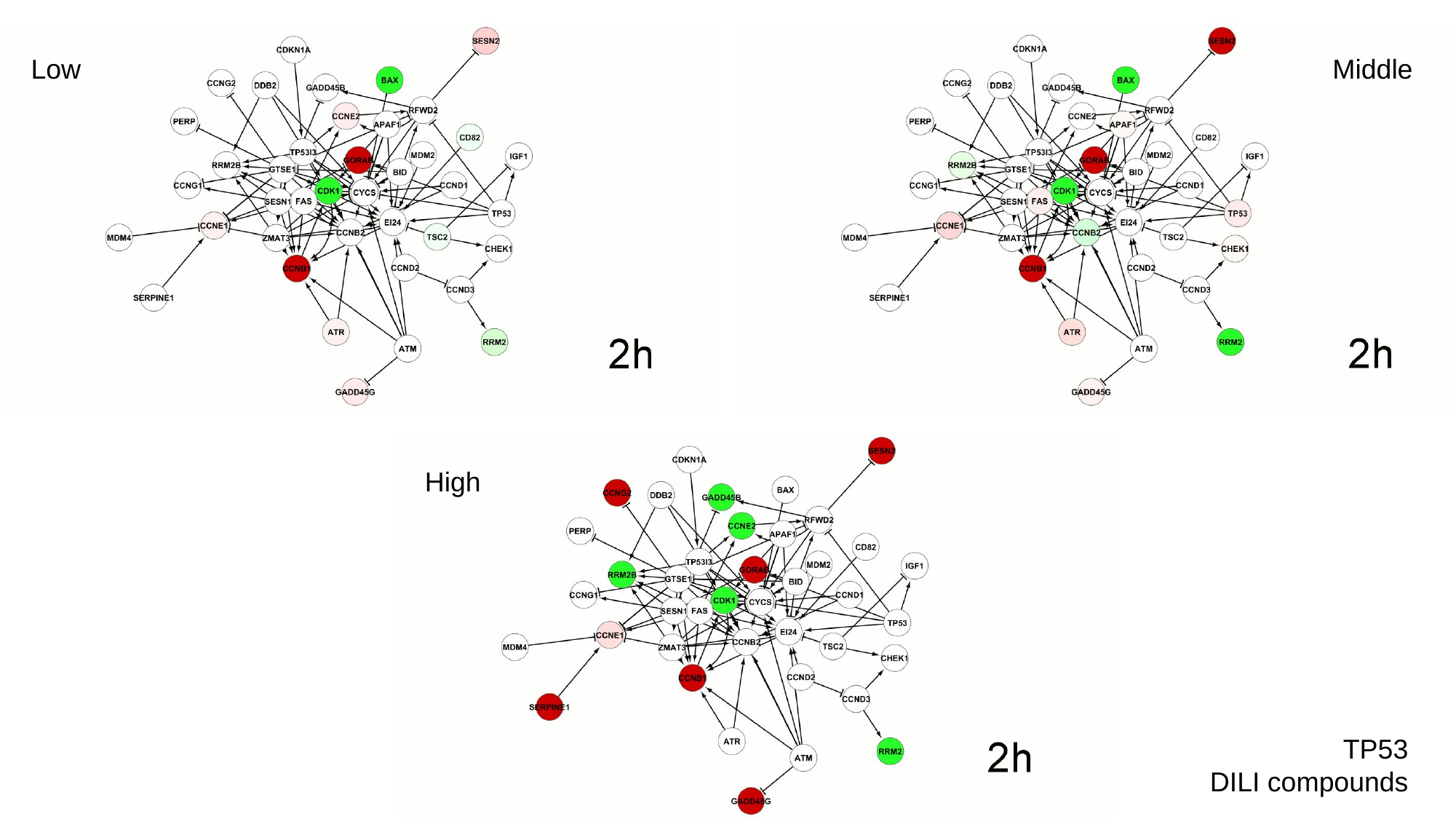

Low
Middle
High
TP53
DILI compounds
